# A Mathematical Model of Diel Activity and Long Time Survival in Phototrophic Mixed-Species Subaerial Biofilms

**DOI:** 10.1007/s11538-024-01348-3

**Published:** 2024-08-28

**Authors:** A. Tenore, F. Russo, J. Jacob, J. D. Grattepanche, B. Buttaro, I. Klapper

**Affiliations:** 1 Department of Mathematics and Applications, University of Naples Federico II, Naples, Italy; 2 U.S. National Park Service, North Atlantic-Appalachian Region, Historic Architecture, Conservation, and Engineering Program, New York, USA; 3 Department of Biology, Temple University, Philadelphia, PA USA; 4 Sol Sherry Thrombosis Research Center, Katz School of Medicine, Temple University, Philadelphia, PA USA; 5 Department of Mathematics, Temple University, Philadelphia, PA USA

**Keywords:** Subaerial biofilm, Cyanobacteria, Heterotrophs, Water activity, Mathematical models

## Abstract

Subaerial biofilms (SAB) are intricate microbial communities living on terrestrial surfaces, of interest in a variety of contexts including cultural heritage preservation, microbial ecology, biogeochemical cycling, and biotechnology. Here we propose a mathematical model aimed at better understanding the interplay between cyanobacteria and heterotrophic bacteria, common microbial SAB constituents, and their mutual dependence on local environmental conditions. SABs are modeled as thin mixed biofilm-liquid water layers sitting on stone. A system of ordinary differential equations regulates the dynamics of key SAB components: cyanobacteria, heterotrophs, polysaccharides and decayed biomass, as well as cellular levels of organic carbon, nitrogen and energy. These components are interconnected through a network of energetically dominant metabolic pathways, modeled with limitation terms reflecting the impact of biotic and abiotic factors. Daily cylces of temperature, humidity, and light intensity are considered as input model variables that regulate microbial activity by influencing water availability and metabolic kinetics. Relevant physico-chemical processes, including pH regulation, further contribute to a description of the SAB ecology. Numerical simulations explore the dynamics of SABs in a real-world context, revealing distinct daily activity periods shaped by water activity and light availability, as well as longer time scale survivability conditions. Results also suggest that heterotrophs could play a substantial role in decomposing non-volatile carbon compounds and regulating pH, thus influencing the overall composition and stability of the biofilm.

**Supplementary Information:**

The online version contains supplementary material available at 10.1007/s11538-024-01348-3.

## Introduction

Subaerial biofilms (SABs) are microbial communities composed of microorganisms embedded in a matrix of extracellular polymeric substances (EPS), and established on solid substrates in contact with the atmosphere. The subaerial environment is often a stark one, so that the development and even presence of microbial communities is already intriguing. Nevertheless, SABs are widespread in nature, including in extreme environments such as arid desert and polar regions, and play a role in many contexts large and small, from climate change and biogeochemical cycles to monument discoloration (Villa et al. 2016). However, knowledge of SAB ecosystems and the factors contributing to their structure and function, and persistence, is still limited. This is at least in part due to difficulties with studying SABs in the field and of replicating them in the laboratory. The goal here, then, is to present a basic, *in silico* SAB model for the purposes of studying basic ecological and physico-chemical SAB issues.

Stone SAB communities exhibit reduced microbial diversity compared to other natural microbial communities (Gorbushina and Broughton 2009), possibly due to the often extreme and fluctuating conditions characterizing stone surface, in terms of nutrients, water availability, temperature, humidity, light, and exposure to wind (Viles and Cutler 2012). These circumstances, though, also make SAB systems attractive mathematical targets; the likelihood of strong coupling between community and physico-chemical environment makes SABs difficult to study in controlled laboratory settings, while their relative simplicity in comparison to many microbial systems makes them more amenable to modeling. As such, SAB models can be of broader interest by serving as one example from which to build models of more complex environmental microbial communities.

A primary factor for the development and maintenance of SABs is unquestionably the availability of liquid water, essential for microbial life as both a solvent and a metabolite. In subaerial environments, liquid water availability often fluctuates significantly due to rainfall and evaporation/condensation events. Periods of poor water conditions pose a significant challenge to microbial life (Zakharova et al. 2013), even though it is well documented in the literature that some SAB microorganisms are capable of surviving long periods of dormancy until water conditions become favorable for metabolic activities (Gorbushina and Krumbein 2000). Even in the absence of rain, though, it is thought that water can be induced to form thin liquid layers on subaerial surfaces via hydrophilic and hygroscopic SAB components (Gorbushina and Broughton 2009; Tenore et al. 2023a) in response to local temperature, humidity, and wind conditions. The EPS matrix, for example, has been reported to play an important role in desiccation tolerance by regulating water loss and absorption (Billi and Potts 2002; Zammit et al. 2011), effectively acting as a reservoir for water and stabilizing desiccation-tolerant enzymes and molecules (Villa and Cappitelli 2019). In any case, experimental studies have observed cell viability in cyanobacterial SABs at relative humidity as low as, approximately, 70% (Villa et al. 2015), implying maintenance of liquid water at this level of relative humidity. Note though that liquid water alone is not sufficient. Rather, water activity—accounting for the amount of free, liquid water available for microbial growth and metabolic activity—is likely to be a critical factor governing the dynamics within SABs (Tenore et al. 2023a).

The self-sustainability of SABs may be further aided by the interplay among the various microbial species inhabiting these ecosystems. SAB microorganisms maintain close proximity, possibly fostering a network of interactions that can serve as a mechanism for resisting physical and nutritional challenges (Vázquez-Nion et al. 2016). Different trophic species are postulated to perform specific vital functions within this oligotrophic microbial society (Villa et al. 2016), possibly exchanging beneficial metabolites and essential nutrients (Paerl et al. 2000). Quantifiable interaction within lab models of stone SABs has in fact been observed between cyanobacteria and heterotrophic bacteria (Villa et al. 2015, 2016). Cyanobacteria are primary producers, capable of using sunlight to produce energy through photosynthesis, while heterotrophs are organisms that rely on organic compounds produced by other organisms as a source of carbon and energy (Cole et al. 2014). This partnership may contribute to the overall stability and functioning of the SAB ecosystem, a possibility which is investigated here.

In recent decades, numerous mathematical models have been proposed to describe the evolution of biofilm systems (Mattei et al. 2018) and explore the ecological dynamics and microbial interplay among photosynthetic and heterotrophic microorganisms (Russo et al. 2023; Tenore et al. 2021, 2023b; Wolf et al. 2007). However, these models are primarily designed for submerged biofilms, where water is generally not a limiting factor and explicitly considered in only a few works (Polizzi et al. 2017, 2022). Additionally, the transport properties of substrates and byproducts differ significantly from those in the SAB environment. Hence, these models are not well suited for characterizing the specialized metabolic networks and the microbial interactions that occur within SABs. In this context, we propose here a new mathematical model aimed at describing the ecology and metabolic dynamics of stone SABs, including important biotic and abiotic factors. In accordance with observations (Tenore et al. 2023a), SABs are modelled as thin mixed biofilm-liquid water layers sitting on stone. A system of ordinary differential equations (ODEs) regulates the dynamics of what appear to be key SAB components, namely cyanobacteria, heterotrophs, polysaccharides and decayed biomass, as well as cellular levels of organic carbon, nitrogen and energy. These components are interconnected through a simplified network of metabolic pathways. Temperature, humidity, and light intensity are considered as input model variables that regulate microbial activity by influencing water availability and metabolic kinetics. In particular, we monitor SAB water activity by following the approach proposed in Tenore et al. (2023a). Relevant physico-chemical processes involved in the SAB are also considered, including solubilization of oxygen, solubilization and hydration of carbon dioxide, as well as regulation of pH levels through charge balance. Notably, outdoor SABs are particularly subject to variations in environmental conditions, which we include in the model and argue to have important consequences for SAB function.

The paper is organized as follows: Sect. 2 focuses on the description of phototrophic SAB ecology, providing an overview of the primary producers, the heterotrophs, and basic pertinent physics and chemistry; Sect. 3 describes a mathematical model of those components, including assumptions, variables, kinetic rates and equations; Sect. 4 illustrates the model setup and presents computational studies; Sect. 5 discusses and concludes the work.

## Ecology of SABs

**Fig. 1:**
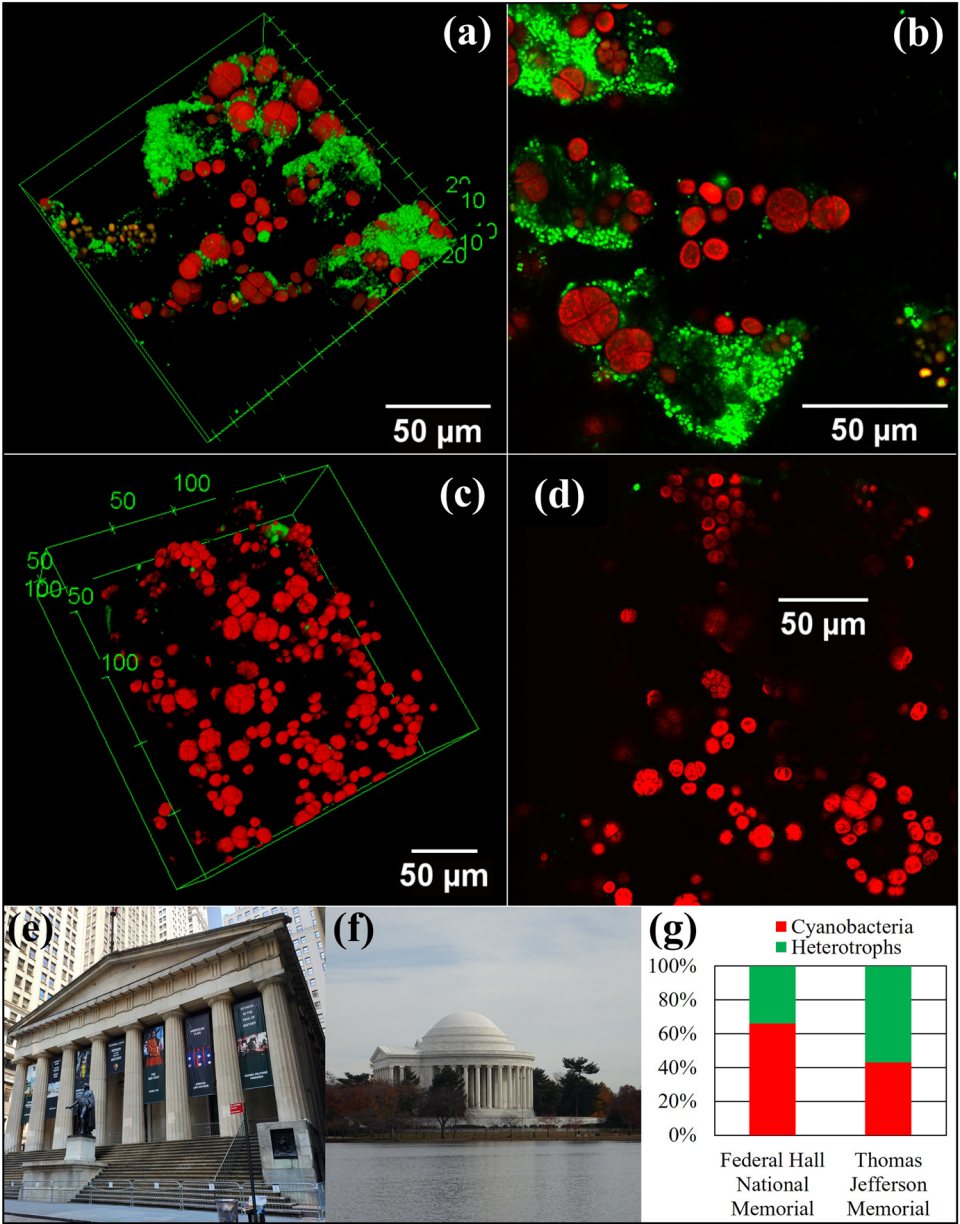
3-dimensional (**a** and **c**) and 2-dimensional (**b** and **d**) confocal views of samples extracted from subaerial biofilms (SABs) inhabiting the marble roofs of the Thomas Jefferson Memorial (**a** and **b**—length and width are 150 $\upmu$m, height is 63 $\upmu$m) and Federal Hall National Memorial (**c** and **d**—length and width are 250 $\upmu$m, height is 79 $\upmu$m). (Red) cyanobacterial autoflorescence; (green) florescent DNA stain. Front views of Federal Hall National Memorial (**e**) and the Thomas Jefferson Memorial (**f**). Relative contribution of cyanobacteria (in red) and heterotrophs (in green) for SAB communities at Federal Hall National Memorial and the Thomas Jefferson Memorial assessed by 16S rRNA gene RNA amplicon sequencing (**g**) (Color figure online)

Based on the literature (Villa et al. 2015, 2016; Tenore et al. 2023a), cyanobacteria and heterotrophic bacteria appear to be common microbial constituents in stone SABs. This statement is also supported by data from samples we collected on the marble roofs of the Thomas Jefferson Memorial in Washington D.C. (construction completed in 1943) and Federal Hall National Memorial in New York City (construction completed in 1842), see Fig. 1. Specifically, we observed, at both sites, the presence of microbial colonies consisting of both cyanobacteria (in red) and heterotrophic bacteria (in green), Fig. 1a, b, as well as, less commonly, colonies composed exclusively of cyanobacteria, Fig. 1c, d.

Consequently, we propose a mathematical model aimed at describing the basic ecology and metabolic dynamics of stone SABs, aimed at exploring the roles of cyanobacteria and heterotrophs in the ecosystem. We note that, while samples are observed to include several species of cyanobacteria as well as a number of different heterotrophic species, to simplify we group the community into two functional classes, or guilds, namely cyanobacteria and heterotrophs. These groups, and their interactions, are discussed in more detail below. Roughly, though, cyanobacteria are the primary producers and heterotrophs are consumers of cyanobacterial byproducts.

**Fig. 2:**
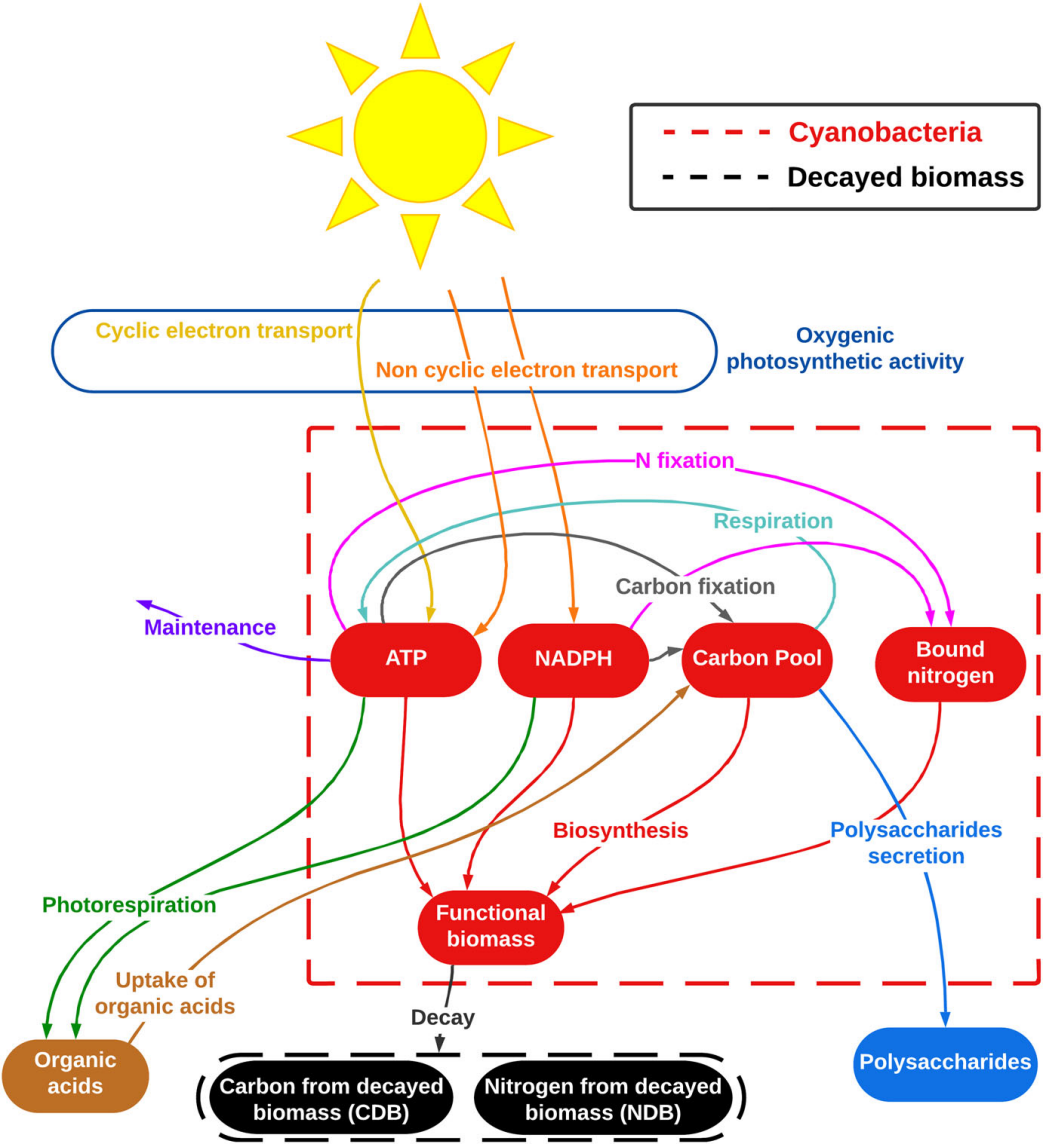
Schematic representation of the model cyanobacteria metabolic network. Dashed boxes represent boundaries between cell insides and outsides. Solid shapes represent SAB components. (Red) cyanobacterial constituents: functional biomass, stored organic carbon, bound nitrogen, ATP, and NADPH levels. (Blue) polysaccharides, (orange) dissolved organic acids, (black) carbon and nitrogen from decayed biomass. Arrows depict metabolic processes. Colors of the arrows indicate different metabolic processes, as labeled in the diagram (Color figure online)

**Fig. 3:**
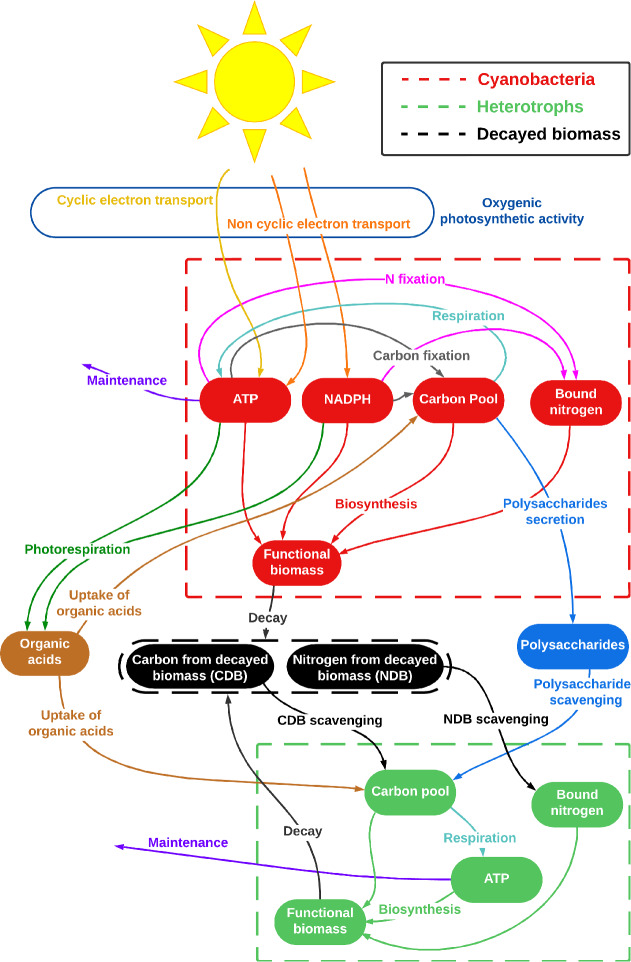
Schematic representation of the SAB metabolic network, adding heterotrophs to the cyanobacterial model in Fig. 2. Dashed boxes represent boundaries between cell insides and outsides. Solid shapes represent SAB components. (Red) cyanobacterial constituents: functional biomass, stored organic carbon, bound nitrogen, ATP, and NADPH levels. (Green) heterotrophic constituents: functional biomass, stored organic carbon, bound nitrogen and ATP level. (Blue) polysaccharides. (Orange) dissolved organic acids. (Black) carbon and nitrogen from decayed biomass. Arrows depict metabolic processes. Colors of the arrows indicate different metabolic processes, as labeled in the diagram (Color figure online)

In accordance with (Tenore et al. 2023a), our model defines an SAB as a mixed biofilm-liquid water layer with a constant thickness of a few tens of microns. Due to its thinness, this layer is assumed to be homogeneous, i.e., with no spatial dependence in substrate concentrations and light intensity across its depth. Environmental conditions, in the form of temperature and humidity, influence the availability of water in the SAB and, consequently, the fluxes of nutrients and energy. Notably, the model accounts for SAB water activity, which impacts metabolic activity and thereby regulates the amounts of carbon and nitrogen, as well as energy level in microbial cells. Diagrams presented in Figs. 2 and 3 illustrate all metabolic pathways included, and serve as a visual guide to the interplay of biochemical processes detailed and modeled in the following sections.

### Cyanobacteria

Cyanobacteria are primary producers of organic carbon from CO$_2$ through oxygenic photosynthesis, and in some cases are also able to fix N$_2$ into bound nitrogen (Gorbushina and Broughton 2009; Steunou et al. 2008). Some cyanobacterial species have additional distinctive properties useful for subaerial environments (Gorbushina and Broughton 2009): adaptation to a wide range of solar radiation intensity; tolerance to water stress conditions; abundant EPS production. It is believed, and we hypothesize here, that cyanobacteria incorporate carbon and nitrogen into the SAB ecosystem, establishing a vital resource for other bacterial species (Cole et al. 2014; Gorbushina and Broughton 2009; Villa et al. 2015).

The diagram presented in Fig. 2 illustrates the metabolic network of the cyanobacteria model. A single microbial cell (used as a representative unit for the entire population) is defined by the dashed red box and comprises diverse intracellular components, represented as solid red shapes. Interactions among these components occur through biochemical processes, as indicated by labeled arrows. The cell also interacts with the extracellular environment by absorbing photons and volatile inorganic compounds, while releasing organic products into the SAB environment.

Oxygenic photosynthetic activity, which is at the foundation of many SABs, can be divided into two separate processes (Rastogi 2021): the light-dependent phase, during which light energy is used to generate ATP (energy storage molecules) and NADPH (reduced electron carriers), and the light-independent phase, during which ATP and NADPH are oxidized to fix inorganic carbon. The light-dependent phase mostly relies on non-cyclic photophosphorylation: electrons removed from water pass through photosystem II (PSII) and then photosystem I (PSI) and end up in NADPH. In this pathway, photons are captured by both PSI and PSII, and ATP is also produced. Additionally, cyanobacteria are able to carry out an alternative electron transport pathway called cyclic photophosphorylation in which electrons cycle repeatedly through PSI without involving PSII. ATP is generated but not NADPH in this case. Typically, the non-cyclic electron transport chain represents the primary and more relevant pathway, while the cyclic one provides an extra contribution of ATP to balance the metabolic ATP to NADPH ratio (Zhang et al. 2023). (Note that the reducing power of NADPH specifically is needed for carbon or nitrogen fixation; ATP is a general energy source.) However, electron flux through PSII and PSI need not balance. For example, PSII can be damaged under water or light stress conditions, compromising the successful outcome of the non-cyclic photophosphorylation (Harel et al. 2004; Ohad et al. 2005). Under some circumstances, cyclic photophosphorylation can become predominant: the cell no longer produces NADPH for biosynthesis but ATP is still generated to fulfill energy requirements for maintenance and survival (Fork and Herbert 1993; McKinlay et al. 2020).

ATP and NADPH generated in the light-dependent phase can be used in the light-independent phase of photosynthesis, also known as the dark reaction, for carbon fixation. This pathway leads to the formation of a fixed carbon pool which supplies organic carbon for subsequent metabolic activities (Kromkamp 1987). However, in the process, oxygen also competes for the same binding sites as inorganic carbon. Where an oxygen molecule successfully binds, it results in the production and, possibly, release of organic waste compounds. This phenomenon is commonly referred to as photorespiration (Falkowski and Raven 2013). The released organic compounds may be reabsorbed and used for respiration (Tolbert and Zill 1956) or, alternatively, can serve as a nutrient supply for other microorganisms (Cole et al. 2014).

In addition to photosynthesis and photorespiration, we include a nitrogen fixation pathway (converting atmospheric nitrogen (N$_2$) into usable forms) in the model, thus alleviating the demand for external, reduced nitrogen and also accounting for a significant energy demand (Steunou et al. 2008). Carbon and nitrogen fixation processes enable the establishment of reserves of organic compounds, which in turn support subsequent metabolic activities. Specifically, stored organic carbon and bound nitrogen are available for use in biosynthesis of new cells. Cyanobacteria can also use stored organic carbon for producing ATP through respiration during nighttime (Armstrong 2014) and secreting polysaccharides, for example in response to osmotic stress (Costa et al. 2018; Macedo et al. 2009; Morcillo and Manzanera 2021).

It is important to note that the processes of carbon fixation and biosynthesis are assumed to take place exclusively in the presence of light. While the term “light-independent” may be misleading, as it suggests that this phase can occur without light, it actually refers to reactions involved that do not directly rely on light energy. However, the light-independent phase, as well as biosynthesis processes, rely on several enzymes that are indirectly dependent on the presence of light for their activity (Lonergan 2000). In the absence of light energy, both photosynthesis and some growth processes shut down, and cyanobacteria depend on the organic carbon stored during the day to maintain their fundamental metabolic activities (Smith 1983); through the respiration process, they generate ATP to sustain vital cellular functions.

In addition to production, we hypothesize that maintenance is a relevant energy consuming process in the SAB ecosystems: when bacteria are exposed to high osmotic tensions, such as in environments with low water availability, they experience osmotic stress (Gustafsson et al. 1993). In order to counteract this stress and maintain cellular homeostasis, they need to expend significant amounts of energy (Harris 1981). Failure to meet maintenance energy requirements could lead to cellular decay. We expect that maintenance demands are relatively energy intensive both for cyanobacteria and heterotrophs as a consequence of environmental stresses, and are key parts of our model.

### Heterotrophs

SAB communities, including the ones we consider here, also generally include heterotrophic bacteria. Heterotrophs lack the ability to assimilate inorganic carbon, and rely on organic carbon compounds synthesized by other organisms for their energy and carbon needs (Cole et al. 2014). Consequently, where they appear in stone SABs, such organisms are typically associated with cyanobacteria, as seen for example in Fig. 1a, b. Note that evidence from laboratory experiments (Villa et al. 2015) suggests that the presence of heterotrophs in SABs promotes and accelerates the proliferation of cyanobacteria compared to a mono-species cyanobacterial SAB.

In light of this, we model heterotrophs as a second functional group, or guild, interacting with cyanobacterial byproducts through the network depicted in Fig. 3, where a heterotrophic cell is defined by the dashed green box. Heterotrophs are able to exploit diverse non-volatile carbon and nitrogen sources originating from cyanobacteria (Albertano 2012; El Moustaid et al. 2017; Villa and Cappitelli 2019). These resources include polysaccharide compounds, organic acids, and decayed biomass, which can be broken down into smaller parts in order to accumulate nitrogen and carbon for utilization in metabolic activities. Specifically, heterotrophs may utilize stored organic carbon as an energy source, producing ATP through respiration essential for sustaining maintenance, and also consume stored organic carbon, nitrogen, and ATP in the biosynthesis of new cells. However, their role may extend beyond commensalism. Heterotrophs can have a positive impact on cyanobacteria by scavenging waste products (Cole et al. 2014). Particularly, we hypothesize here that heterotrophs degrade non-volatile organic acids released by cyanobacteria (El Moustaid et al. 2017) and maintain the pH level in the ecosystem within the optimal range for microbial metabolic processes.

### Physics and chemistry

Environmental microbial communities are inherently exposed to the variable physical and chemical stresses imposed by the environment around them. SABs, notably, including the type studied here, are frequently subject to inherently dry conditions. Any microbial cell needs liquid water for its survival, including for those chemical reactions which are part of its metabolic activities. However, liquid water in complex systems with dissolved and undissolved contaminants (e.g., SAB components) is not necessarily easily accessible due to both energetic and entropic conditions.

Availability of water for chemical reactions is typically measured through a water activity coefficient $a_w$, which can be defined as the ratio of the vapor pressure of liquid water in the biofilm to the vapor pressure of pure liquid water at the same temperature and pressure (Tenore et al. 2023a). This coefficient is expressed as a dimensionless number between 0 and 1, where 0 represents no water availability (completely dry) and 1 represents pure liquid water (no contaminants). Water activity in SABs is subject to great variability, affected by rain events and condensation/evaporation mechanisms. Reduced water activity levels cause osmotic stress (effectively, competition for accessible liquid water), thereby increasing the energy requirements for maintaining osmotic balance and cellular homeostasis. Notably, low water activity can inhibit cell metabolism and induce microorganisms into a dormant state or, in some cases, death (Billi and Potts 2002).

In addition to water, the ecology and dynamics of phototroph-based SABs also depends on light, i.e., photons, which serve as the primary energy source for photosynthetic activity, ultimately regulating the overall metabolism of the ecosystem. Though, while light is necessary for the sustainability of SABs, excessive light intensity can induce negative effects on microorganisms, such as photoinhibition and, as well, dehydration via increased temperature (and attendant drop in relative humidity). Indeed, the most challenging conditions of water stress typically align with periods of maximum light intensity.

In the case of SABs, particularly thin ones, we also suggest that volatility (or non-volatility) of particular chemical compounds can be significant; volatile substrates can be easily replenished from the air if available while volatile byproducts can be easily released. Conversely, non-volatile byproducts are essentially trapped. Volatile compounds like O$_2$ and CO$_2$ also play a fundamental role in the ecology of SABs, constituting important components of heterotrophic and phototrophic metabolism. In general, the availability of O$_2$ and CO$_2$ within SABs is regulated by gas transfer and diffusion processes, chemical reactions, and metabolic processes. Note, though, that while O$_2$ and CO$_2$ concentrations in very thin SABs are generally fixed by atmospheric concentrations, they could be locally higher or lower in thicker SABs, where limitations in mass-transport diffusion occur. In particular, the characteristic time for diffusion in a 15 $\upmu$m thick SAB (as considered here) can be estimated to be an order of magnitude lower than those for significant metabolic processing of CO2 and O$_2$. Conversely, these phenomena are found to have similar characteristic time scales for SABs of 50–100 $\upmu$m thickness. Such SABs may as a result develop spatial variations in chemical concentrations and so not be considered to be thin in the sense we use here.

Another source of inorganic carbon within SABs is HCO$_3^-$ (bicarbonate), deriving from chemical equilibrium with CO$_2$ in aqueous solution and hence also impacted by volatility considerations. This equilibrium is influenced by and, in turn, influences the pH levels within the ecosystem. pH plays a role in protein structure, enzyme activity, and regulating the bioavailability of nutrients and trace elements, thereby impacting microbial growth (Jin and Kirk 2018). The acidity or alkalinity of SABs can be also influenced by metabolic processes, such as photosynthesis leading to the consumption of bicarbonate or photorespiration resulting in the production of non-volatile organic acids, as well as by interactions with stone substratum and atmosphere.

## Mathematical model

The narrative of Sect. 2, as illustrated in Figs. 2 and 3 has been translated into a differential equation based model consisting of two guilds (cyanobacteria and heterotrophs), their simplified metabolisms, and the extracellular physical and chemical processes (as in Figs. 2 and 3). Details are presented below and in Appendix A.

To summarize: the model considers cyanobacteria employing both cyclic and non-cyclic electron transport pathways during their light-dependent photosynthetic phase, according to water activity conditions. The subsequent light-independent phase of photosynthesis relies on carbon fixation, resulting in the accumulation of intracellular organic carbon as well as in photorespiration, leading to the release of extracellular waste organic acids. Competition between carbon fixation and photorespiration is described using a branching function as in El Moustaid et al. (2017). Cyanobacteria are also assumed to fix nitrogen from the atmosphere (Grover et al. 2019) and carry out respiration during nighttime (Armstrong 2014). Production of extracellular polysaccharides by cyanobacteria can be controlled by osmotic stress (Macedo et al. 2009; Wu et al. 2021).

The second guild type, heterotrophs, exploit varied sources of organic carbon and nitrogen for the biosynthesis of new cells, respiration and maintenance: polysaccharides matrix, decayed biomass and waste organic acids released by cyanobacteria during photorespiration. Biosynthesis of both cyanobacteria and heterotrophs is regulated by organic carbon, nitrogen, and ATP levels within the cell. Maintenance energy demand is formulated as a decreasing function of water activity, and decay processes are triggered when cells do not have sufficient energy to cover maintenance costs.

We also model the influence of relevant environmental factors on microbial metabolic processes, including water activity, light exposure, pH levels, availability of dissolved inorganic carbon and oxygen. Water activity is modeled as a time-dependent function of input air and stone temperature and humidity conditions, as introduced in Tenore et al. (2023a). Light intensity is accounted as an additional input variable. pH levels are monitored using a charge balance method, while concentrations of volatile compounds are determined at a steady state using Henry’s law.

### Cyanobacteria

The cyanobacteria-only model, consists of the following (five intracellular fields): total functional biomass $M_C(t)$ $[\textrm{mol C}]$, quota of stored organic carbon (SOC) per cell mass $Q_{SOC,C}(t)$ $[{\textrm{mol C}}/{\textrm{mol C}}]$, quota of bound nitrogen (N) per cell mass $Q_{N,C}(t)$ $[\textrm{mol N}/\textrm{mol C}]$, quota of ATP per cell mass $Q_{ATP,C}(t)$ $\ [{\textrm{mol ATP}}/{\textrm{mol C}}]$, and quota of NADPH per cell mass $Q_{NADPH,C}(t)$ $[{\textrm{mol NADPH}}/{\textrm{mol C}}]$, see solid red ellipses in Fig. 2, and (six extracellular fields): polysaccharides (POL) $M_{\textrm{POL}}(t), [\textrm{mol C}]$, carbon decayed biomass (CDB) $M_{\textrm{CDB}}$ $[\textrm{mol C}]$, nitrogen decayed biomass (NDB) $M_{NDB}(t)$ $[\textrm{mol N}]$, total organic acids (TOA) $S_{TOA}(t)$ $[\textrm{mol C}/\textrm{m}^3]$, and anion organic acid (OA$^-$) $S_{OA^-}(t)$ $[\textrm{mol C}/\textrm{m}^3]$, bicarbonate (HCO$_3^-$) $S_{HCO_3}(t)$ $[\textrm{mol C}/{\textrm{m}^3}]$, see remaining solid ellipses in Fig. 2. The abbreviations molC and molN denote moles of carbon and nitrogen, respectively. They are convenient measurement units when tracking carbon and nitrogen balance, as we do here, allowing for example quantification of cellular biomass in terms of its carbon content. The introduction of quotas and the division of the microbial cell into nutrient pools and functional biomass follow the approach outlined in Mairet et al. (2011), Polizzi et al. (2017), Polizzi et al. (2022).

The intracellular fields are governed by the equations

1 $$\begin{aligned} \frac{dM_\textrm{C}}{dt}= &  \sum _{i} K_{\textrm{C},i} \, r_{i}, \end{aligned}$$

2 $$\begin{aligned} \frac{d(M_C Q_{\textrm{SOC}})}{dt}= &  \sum _{i} K_{\textrm{SOC},i} \, r_{i}, \end{aligned}$$

3 $$\begin{aligned} \frac{d(M_C Q_{\textrm{N,C}})}{dt}= &  \sum _{i} K_{\textrm{N},i} \, r_{i}, \end{aligned}$$

4 $$\begin{aligned} \frac{d(M_C Q_{\textrm{ATP,C}})}{dt}= &  \sum _{i} K_{\textrm{ATP},i} \, r_{i}, \end{aligned}$$

5 $$\begin{aligned} \frac{d(M_C Q_{\textrm{NADPH,C}})}{dt}= &  \sum _{i} K_{\textrm{NADPH},i} \, r_{i}, \end{aligned}$$

together with initial conditions $M_C(0)=M_{C,0}$, $Q_{SOC,C}(0)=Q_{SOC,C,0}, Q_{N,C}(0)=Q_{N,C,0}$, $Q_{ATP,C}(0)=Q_{ATP,C,0}$, and $Q_{NADPH,C}(0)=Q_{NADPH,C,0}$. Extracellular fields are governed by the equations

6 $$\begin{aligned} \frac{d(M_{POL})}{dt}= &  \sum _{i} K_{POL,i} \, r_{i}, \end{aligned}$$

7 $$\begin{aligned} \frac{d(M_{CDB})}{dt}= &  \sum _{i} K_{CDB,i} \, r_{i}, \end{aligned}$$

8 $$\begin{aligned} \frac{d(M_{NDB})}{dt}= &  \sum _{i} K_{NDB,i} \, r_{i}, \end{aligned}$$

9 $$\begin{aligned} \frac{d(V S_{TOA})}{dt}= &  \sum _{i} K_{TOA,i} \, r_{i}, \end{aligned}$$

10 $$\begin{aligned} \frac{d(V S_{OA^-})}{dt}= &  \sum _{i} K_{OA^-,i} \, r_{i}, \end{aligned}$$

11 $$\begin{aligned} \frac{d(V S_{HCO_3})}{dt}= &  \sum _{i} K_{HCO_3,i} \, r_{i}, \end{aligned}$$

with initial conditions $M_{POL}(0)=M_{POL,0}$, $M_{CDB}(0)=M_{CDB,0}, M_{NDB}(0)=M_{NDB,0}$, $S_{TOA}(0)=S_{TOA,0}, S_{OA^-}(0)=S_{OA^-,0}$ and $S_{HCO_3}(0)=S_{HCO_3,0}$.

The right-hand sides of (1)–(11) encode rates of metabolic processes, see arrows in Fig. 2, indexed by *i*: parameters $K_{j,i}$ are stoichiometric coefficients for input/output of process *i*, with process rate $r_{i}(t)$. Index *i* ranges over the processes seen in Fig. 2, namely cyclic and non-cyclic electron transport, carbon fixation, nitrogen fixation, photorespiration, uptake of organic acids, respiration, biosynthesis, polysaccharide production and secretion, maintenance. *V* represents the SAB volume, computed as $V = M / \rho$, with $\rho$ denoting the constant microbial density of the SAB, and *M* the total mass of SAB solid-phase components, computed as $M = M_C (1+Q_{SOC,C}) + M_H (1+Q_{SOC,H}) + M_{POL} + M_{CDB}$. (Observe that, as the SAB thickness is constant, any change in volume associated with the variation in mass of the SAB components results exclusively in a change in the area of stone colonized by the SAB.)

Process rate functions are discussed below, with detailed formulations provided in the Appendix A, but note that each process can be limited by a range of factors, including water activity, light conditions, and the availability of carbon, nitrogen, and energy within the ecosystem and/or cells. To assess the impact of each factor *k* on these biological processes, we calculate separate limitation terms $\phi _{k}$, each taking values in the range [0, 1] and write each $r_{i}$ in the form

12 $$\begin{aligned} r_{i} = k_{i} M_{i} f_{i}, \ f_{i} = \min (\phi _1,...,\phi _{k}), \end{aligned}$$

where $k_{i}$ is the maximum rate of the biological process *i*, and $M_{i}$ is the mass of the species carrying out the biological process *i*. Instead of including multiple simultaneous limitations, this approach considers a single limiting factor, preventing the underestimation of rates that might occur when summing the limiting effects of all involved factors (Wolf et al. 2007).

#### Photosynthesis-related rates

The light-dependent phase of photosynthesis is divided into non-cyclic and cyclic electron transport chains (ETC), with flux rates

13 $$\begin{aligned} r_{nc}= &  k_{nc} \ M_C \ f_{C,nc}, \end{aligned}$$

14 $$\begin{aligned} r_{cy}= &  k_{cy} \ M_C \ f_{C,cy,1} + \Delta k_{cy} \ M_C \ f_{C,cy,2}, \end{aligned}$$

where $k_{nc}$ is the maximum non-cyclic photophosphorylation rate, $k_{cy}$ is the basal cyclic photophosphorylation rate; $\Delta k_{cy}$ is the maximum increment of cyclic photophosphorylation rate. In line with the findings outlined in the previous section, the non-cyclic photophosphorylation rate $r_{nc}$ is characterized as an increasing function of water activity. The cyclic photophosphorylation rate $r_{cy}$ is formulated as the sum of two contributions. The first one represents a basal rate that balances the metabolic ATP to NADPH ratio under ideal water conditions $a_w=1$, while the second one increases as water activity decreases, fulfilling the extra ATP requirements for maintenance. Accordingly, under ideal water conditions ($a_w=1$), $r_{nc}$ is at its maximum value, while $r_{cy}$ only results in a basal production of ATP ($f_{C,cy,2} = 0$). For lower $a_w, r_{nc}$ decreases (by decreasing $f_{C,nc}$), while $r_{cy}$ increases (by increasing $f_{C,cy,2}$). However, both photophosphorylation processes slow down until they cease for water activity approaching the minimum viable value ($f_{C,nc}=f_{C,cy,1}=f_{C,cy,2}=0$). Detailed expressions for these processes are provided in the Appendix A. (Water activity $a_w(t)$, which tracks liquid water activity in the biofilm, is derived as a steady-state function of local temperature and relative humidity values, see Sect. 3.3.)

Light intensity *I*(*t*) ($\upmu$E m$^{-2}$ s$^{-1}$), a provided function, can limit at low intensity levels both cyclic and non-cyclic pathways, while inhibiting at high levels the non-cyclic one (causing photodamage to PSII). Due to thinness, *I*(*t*) is assumed to be constant throughout the SAB, that is, shading effects are neglected. Also, non-cyclic photophosphorylation can be limited by NADPH availability, and both pathways can be limited by ATP availability.

Inorganic carbon is fixed in the second, light-independent phase of photosynthesis, in competition with photorespiration. Similarly to El Moustaid et al. (2017), the interplay between carbon fixation $r_{fix}$ and photorespiration $r_{photo}$ rates is modeled by introducing a photorespiration branching function $g_{fix}$

15 $$\begin{aligned} g_{fix} = \frac{1}{1+\gamma \frac{S_{O_2}}{S_{DIC}}}, \end{aligned}$$

where $\gamma$ is the inverse specificity factor, which balances the two process rates according to

16 $$\begin{aligned} r_{fix}= &  g_{fix} \ k_{fix} \ M_C \ f_{C,fix}, \end{aligned}$$

17 $$\begin{aligned} r_{photo}= &  (1 - g_{fix}) \ k_{fix} \ M_C \ f_{C,fix}, \end{aligned}$$

where $k_{fix}$ is the maximum carbon fixation rate.

These processes require inorganic carbon, and thus $f_{C,fix}$ depends on $S_{DIC}$, the concentration of dissolved inorganic carbon *DIC*, determined by summing the concentrations of dissolved carbon dioxide and bicarbonate ions, i.e., $S_{DIC} = S_{CO_2} + S_{HCO_3^-}$. Steady-state dynamics are assumed for volatile substrates (i.e., carbon dioxide $S_{CO_2}$ and dissolved oxygen $S_{O_2}$, see Sect. 3.3), supposing that substrate diffusion and gas-transfer processes occur much more rapidly than variations in biofilm metabolic activities and environmental conditions.

Carbon fixation and photorespiration are also regulated by cellular quotas of ATP, NADPH and stored organic carbon, light availability, and pH. In particular, they are constrained by availability of ATP, NADPH, and high levels of stored organic carbon within the cell. Also, both rates approach zero in the absence of light ($I(t)=0$) or when pH values are outside the viable range.

We also assume that SAB cyanobacteria invest part of their ATP energy for fixing atmospheric-derived N$_2$ into bound nitrogen (Grover et al. 2019):

18 $$\begin{aligned} r_{Nfix} = k_{Nfix} \ M_C \ f_{C,Nfix}, \end{aligned}$$

where $k_{Nfix}$ is the maximum nitrogen fixation rate. Limitation factor $f_{C,Nfix}$ is a function of nitrogen, ATP, and NADPH availability, as well as water activity. As a note, sequencing results for the Thomas Jefferson Memorial and Federal Hall National Memorial samples are consistent with the ability of SAB cyanobacteria to fix atmospheric nitrogen.

#### Synthesis-related rates

In the presence of light, stored organic carbon and bound nitrogen can be used for biosynthesis of new cells at rate

19 $$\begin{aligned} r_{C, growth} = k_{growth,C} \ M_C \ f_{C,growth}, \end{aligned}$$

where $k_{growth,C}$ is the maximum growth rate for cyanobacteria. Limitation factor $f_{C,growth}$ depends on available intracellular resources, namely stored organic carbon, bound nitrogen, ATP and NADPH.

Secretion of polysaccharides by cyanobacteria is formulated as

20 $$\begin{aligned} r_{POLprod} = k_{pol} \ M_C \ f_{C,pol}, \end{aligned}$$

where $k_{pol}$ is the maximum polysaccharide production rate. Limitation factor $f_{C,pol}$ depends on organic carbon availability as well as water activity. The influence of water activity on polysaccharide production has been modeled similarly to its influence on cyclic photophosphorylation: as water activity decreases, polysaccharide production intensifies in response to osmotic stress. However, as water activity continues to decrease, production slows down until it stops for water activity approaching the minimum viable value.

#### Respiration-related rates

In the absence of light energy, cyanobacteria may use stored organic carbon for respiration, generating ATP to sustain vital cellular functions at rate

21 $$\begin{aligned} r_{C,resp} = k_{resp} \ M_C \ f_{C,resp}, \end{aligned}$$

where $k_{resp}$ is the maximum respiration rate. Limitation factor $f_{C,resp}$ depends on organic carbon and ATP availability, as well as light intensity and water activity.

Additionally, we consider the reabsorption of organic acids by cyanobacteria during the night, which counterbalances charge movement due to respiration (Tolbert and Zill 1956):

22 $$\begin{aligned} r_{C,TOA \ uptake} = k_{uptake} \ M_C \ f_{C,uptake}, \end{aligned}$$

where $k_{uptake}$ is the maximum rate of organic acids uptake. Limitation factor $f_{C,uptake}$ depends on organic carbon interior and exterior to the cell, as well as light intensity and water activity.

#### Maintenance and decay-related rates

SABs are, in many cases, only marginally viable and near a steady state. Hence we hypothesize that maintenance activity is an important aspect of their energy budget. Maintenance rate

23 $$\begin{aligned} r_{C,main} = M_C \ f_{C,main}, \end{aligned}$$

is assumed, through $f_{C,main}$, to depend on water activity, ATP availability and the ATP production rate (in cyanobacteria). Under adequate concentration and production of ATP, $f_{C,main}$ is equal to the total maintenance requirement $f^*_{main}$, increasing with decreasing $a_w$ (with a linear dependence). Conversely, when the ATP concentration and production do not meet the total maintenance requirement, $f_{C,main}<f^*_{main}$ and is equal to the overall ATP production rate $\omega _C$, under the assumption that ATP produced is totally allocated to maintenance.

When maintenance rate is insufficient, e.g., ATP level in the cell does not meet the total maintenance energy requirement, cellular decay occurs:

24 $$\begin{aligned} r_{C,d} = M_C \ f_{C,d}, \end{aligned}$$

where limitation factor $f_{C,d}$ is assumed to be proportional to the missing energy rate

25 $$\begin{aligned} \gamma _C=\frac{\omega _C}{f^*_{main}}. \end{aligned}$$

It should be noted that an unfavorable water activity value has a dual negative impact on ecosystem self-sustainability. Firstly, it leads to a high energy requirement for maintenance, and secondly, it slows down/inhibits the ATP production processes.

### Heterotrophs

We extend the cyanobacterial SAB to a mixed species, cyanobacteria-heterotroph SAB, see solid green ellipses in Fig. 3, adding four intracellular heterotroph fields: total functional biomass $M_H(t)$ $[\textrm{mol C}]$, quota of stored organic carbon (SOC) per cell mass $Q_{SOC,H}(t)$ $[{\textrm{mol C}}/{\textrm{mol C}}]$, quota of bound nitrogen (N) per cell mass $Q_{N,H}(t)$ $[{\textrm{mol N}}/{\textrm{mol C}}]$, and quota of ATP per cell mass $Q_{ATP,H}(t)$ $[{\textrm{mol ATP}}/{\textrm{mol C}}]$.

Heterotroph intracellular fields are governed by differential equations of the same form as (1)–(5), namely

26 $$\begin{aligned} \frac{dM_H}{dt}= &  \sum _{i} K_{H,i} \, r_{i}, \end{aligned}$$

27 $$\begin{aligned} \frac{d(M_H Q_{SOC,H})}{dt}= &  \sum _{i} K_{SOC,i} \, r_{i}, \end{aligned}$$

28 $$\begin{aligned} \frac{d(M_H Q_{N,H})}{dt}= &  \sum _{i} K_{N,i} \, r_{i}, \end{aligned}$$

29 $$\begin{aligned} \frac{d(M_H Q_{ATP,H})}{dt}= &  \sum _{i} K_{ATP,i} \, r_{i}, \end{aligned}$$

together, again, with initial conditions $M_H(0)=M_{H,0}$, $Q_{SOC,H}(0)=Q_{SOC,H,0}, Q_{N,H}(0)=Q_{N,H,0}$, and $Q_{ATP,H}(0)=Q_{ATP,H,0}$. Processes encoded in the right-hand sides of (26)–(29) are discussed below with detailed rate expressions provided in Appendix A.

#### Uptake rates

Heterotrophs carry out their metabolic activities by exploiting different nutrient sources, here grouped as polysaccharide matrix and organic acids produced by cyanobacteria, and decayed biomass. (Note that the scavenging of polysaccharides and decayed biomass offers opportunities for heterotrophs to break down complex organic molecules and thereby accumulate nitrogen and carbon.) Scavenging processes occur, respectively, at rates

30 $$\begin{aligned} r_{POL \,scav}= &  k_{scav} \ M_H \ f_{H,POL \,scav}, \end{aligned}$$

31 $$\begin{aligned} r_{CDB \,scav}= &  k_{scav} \ M_H \ f_{H,CDB \,scav}, \end{aligned}$$

32 $$\begin{aligned} r_{NDB \,scav}= &  k_{scav} \ M_H \ f_{H,NDB \,scav}, \end{aligned}$$

where $k_{scav}$ is the maximum scavenging rate. In addition to polysaccharide and decayed biomass availability, these process are also subject to water activity limitation. Heterotrophs are also assumed able to uptake dissolved organic acids released by cyanobacterial photorespiration at rate

33 $$\begin{aligned} r_{H,TOA \ uptake} = k_{uptake} \ M_H \ f_{H,uptake}, \end{aligned}$$

again subject to water activity limitation.

#### Synthesis and respiration rates

Stored organic carbon is invested by heterotrophs into respiration and/or biosynthesis, based on the availability of bound nitrogen $Q_{N,H}$ and ATP $Q_{ATP,H}$:

34 $$\begin{aligned} r_{H,resp}= &  k_{resp} \ M_H \ f_{H,resp}, \end{aligned}$$

35 $$\begin{aligned} r_{H,growth}= &  k_{growth,H} \ M_H \ f_{H,growth}, \end{aligned}$$

where $k_{growth,H}$ is the maximum growth rate of heterotrophs.

#### Maintenance and decay rates

Kinetics of maintenance and decay have been modeled similarly as for cyanobacteria, with maintenance and decay rates

36 $$\begin{aligned} r_{H,main}= &  M_H \ f_{H,main}, \end{aligned}$$

37 $$\begin{aligned} r_{H,d}= &  M_C \ f_{H,d}. \end{aligned}$$

Under sufficient ATP concentration and production (in heterotrophs), $f_{H,main}$ matches the total maintenance requirement $f^*_{main}$, inversely proportional to $a_w$, and $f_{H,d}$ is null. When ATP is insufficient, $f_{H,main}$ is equal to the overall ATP production rate $\omega _H$, and $f_{H,d}$ is proportional to the missing energy rate

38 $$\begin{aligned} \gamma _H=\frac{\omega _H}{f^*_{main}}. \end{aligned}$$

### Physico-chemical processes

Since SAB depth is on the order of a few tens of microns, the time scale of gas-transfer processes can be assumed significantly shorter than time scales of biological processes. Consequently, liquid-phase concentrations of volatile compounds, such as oxygen O$_2$ and carbon dioxide CO$_2$, can be estimated to be at steady state using Henry’s law. Detailed formulations are available in the Appendix A.

The pH level in the SAB is monitored through a charge balance (refer to the Appendix A for detailed expressions) that considers the following charged compounds, expressed in concentrations:

- HCO$_3^-$ formed as a result of carbon dioxide dissolution in water.
- Anion organic acids OA$^-$ resulting from the dissociation of organic acids OA released during photorespiration of cyanobacteria.
- Anion An and cation Cat compounds with no consumption or reaction terms.
- Hydrogen ions H$^+$ and hydroxide ions OH$^-$ dissolved in water.

When carbon dioxide CO$_2$ is dissolved in water, it undergoes reactions that lead to the formation of bicarbonate ions HCO$_3^-$ and hydrogen ions H$^+$:

$$\begin{aligned} {\hbox {CO}_2 + \hbox {H}_2\hbox {O}}&\rightleftharpoons {\hbox {H}_2\hbox {CO}_3} \\ {\hbox {H}_2\hbox {CO}_3}&\rightleftharpoons {\hbox {HCO}_3^- + \hbox {H}^+} \end{aligned}$$

Although H$_2$CO$_3$ is a relatively strong acid ($pK_{a,H2CO3} = 3.5$), the equilibrium coefficient for CO$_2$/H$_2$CO$_3$ is very large, that is CO$_2 >>$ H$_2$CO$_3$, and CO$_2$ can be taken as the effective acid (Batstone et al. 2002). These dissolution reactions result in the formation of hydrogen ions, and affect the pH of the water. The concentration of HCO$_3^-$ is dependent on the acid–base rate

39 $$\begin{aligned} r_{HCO_3^-,eq} = k_{AB,CO_2} \bigg (CO_{2,sat} - \frac{S_{H^+} \ S_{HCO_3^-}}{K_{a,CO_2}}\bigg ), \end{aligned}$$

where $k_{AB,CO_2}$ and $K_{a,CO_2}$ are the rate constant and dissociation constant of CO$_2$ hydrolysis. Dissociation of HCO$_3^-$ into CO$_3^{2-}$ is neglected in this model because it is only relevant for very high pH values ($pK_{a,HCO_3^-} = 10.33$).

Similarly, an acid–base reaction governs the equilibrium between organic acid and its conjugate base

$$\begin{aligned} \hbox {OA}&\rightleftharpoons \hbox {OA}^{-} + \hbox {H}^{+} \end{aligned}$$

and the concentration of anion organic acids OA$^-$ is dependent on the acid–base rate

40 $$\begin{aligned} r_{OA^-,eq} = k_{AB,OA} (S_{H^+} \ S_{OA^-} - K_{a,OA} S_{OA}). \end{aligned}$$

Substituting $$S_{OA} = S_{TOA} - S_{OA^-}$$ yields

41 $$\begin{aligned} r_{OA^-,eq} = k_{AB,OA} (S_{OA^-} (K_{a,OA} + S_{H^+}) - K_{a,OA} S_{TOA}), \end{aligned}$$

where $k_{AB,OA}$ and $K_{a,OA}$ are the rate and dissociation constants for OA hydrolysis.

Following the physically-based approach introduced in Tenore et al. (2023a), we model the SAB water activity as a time dependent function of air and stone temperature and humidity conditions (more details are provided in Appendix A):

42 $$\begin{aligned} a_w = RH_a e^{\big (\frac{b T_a}{c+T_a}\big )}e^{\big (-\frac{b T^*}{c+T^*}\big )}, \end{aligned}$$

where $T_a$ and $RH_a$ denote the ambient air temperature and humidity, *b* and *c* are coefficients determined experimentally. $T^*$ is the temperature at the interface biofilm-boundary layer.

We assume the mixed biofilm-liquid water layer to be in contact with the atmosphere, and sitting on an impermeable flat stone, and consider 1-D steady-state diffusive heat transport. Then, $T^*$ can be readily computed, see Tenore et al. (2023a), to obtain

43 $$\begin{aligned} T^* = \frac{T_a k_a \lambda + T_s \delta k_b}{\lambda k_a+\delta k_b}, \end{aligned}$$

where $T_s$ is the stone temperature and $k_a$ and $k_b$ are the thermal diffusivities of air and SAB, respectively, $\lambda$ is the fixed SAB thickness, $\delta$ is the thickness of the thermal boundary layer, that is the distance from the stone surface to the point where the flow temperature has essentially reached the ambient air temperature.

## Analysis of numerical results

Simulations have been conducted to illustrate the behavior and dynamics of the SAB model. In this section, we describe numerical methods and present results.

### Model setup for numerical simulations

Model equations were integrated using a MATLAB code based on the solver ’ode15s’. See Appendix B, Table 1, for initial conditions for the Eqs. (1)–(11) and (26)–(29). Model parameters are reported in Appendix B (see Tables 3 and 4) together with a discussion of parameter choices. Environmental conditions are set (see Appendix B, Table 2), based on a data collection campaign on the marble roof of the portico at the Thomas Jefferson Memorial (Tenore et al. 2023a). In particular, we consider a representative daily profile for each environmental variable, repeating periodically throughout the simulation time. Using this data, we compute a 24 h periodic SAB water activity $a_w$ (Tenore et al. 2023a), which is used to regulate the metabolic network of the system. SAB thickness $\lambda$ has been set at 15 $\upmu$m, in accordance with experimental observations (Tenore et al. 2023a). This model setup leads to an initial transient phase, followed by the establishment of a periodic state where the composition of the biofilm, i.e., all biofilm-specific quantities, exhibits daily periodic profiles. The transient time is due to the arbitrariness of the initial conditions and the fact that the model community is close to marginal, so that the approach to the final state can be slow. In actuality, we are focused here on communities that have already passed their initial transient condition. The initial transients seen in the computations then are not relevant, although they do suggest that a new colony may take considerable time to establish itself.

Note that the presented model does not attempt to predict SAB thickness; in fact, the determinative mechanisms are unclear, and may involve, for example, mechanical factors that are not included in the model. Also, the values for inert anion and cation concentrations $S_{An}$ and $S_{Cat}$ are both set to zero in computations below, as we do not have data from the study sites to characterize them. However, these compounds are likely present in reality, potentially causing fluctuations in pH levels and impacting the overall metabolic network. We do not explore this possibility here.

### Computational results

Three numerical studies are presented. The first illustrates a representative case based on the environmental profile of a typical summer day at the Thomas Jefferson Memorial for which we investigate the development of a cyanobacteria-heterotroph SAB under diel periodic environmental conditions and water activity, and examine how these factors impact the metabolic pathways of the ecosystem. Notably, most metabolic activity is predicted by the model to be confined to relatively brief intervals of the day depending on water activity and light availability.

Next, a second study explores the role of heterotrophs in a mixed species SAB ecosystem (under the same diel environmental conditions), indicating a significant influence on organic acid dynamics, pH regulation, and the overall composition of the biofilm. Specifically, this study suggests that heterotrophs degrade organic acids, thus stabilizing pH at levels amenable for cyanobacterial growth. On the other hand, in the absence of heterotrophs, pH levels that hinder cyanobacterial growth are predicted, leading instead to an increased abundance of polysaccharides in the ecosystem.

The third study extends across the four seasons of the year using seasonally averaged profiles that emphasize the SAB’s acclimation to diverse environmental conditions. Results highlight the interplay between temperature, water activity, and microbial dynamics, offering insights into the resilience and metabolic strategies of SAB ecosystems across diverse scenarios. In particular, summer and spring present challenges for SAB microorganisms due to limited water availability and high osmotic tension, leading to significant decay processes. In contrast, winter and autumn maintain comparatively elevated water activity levels throughout the day, sustaining efficient metabolic activities.

#### Numerical study 1: Representative case

**Fig. 4:**
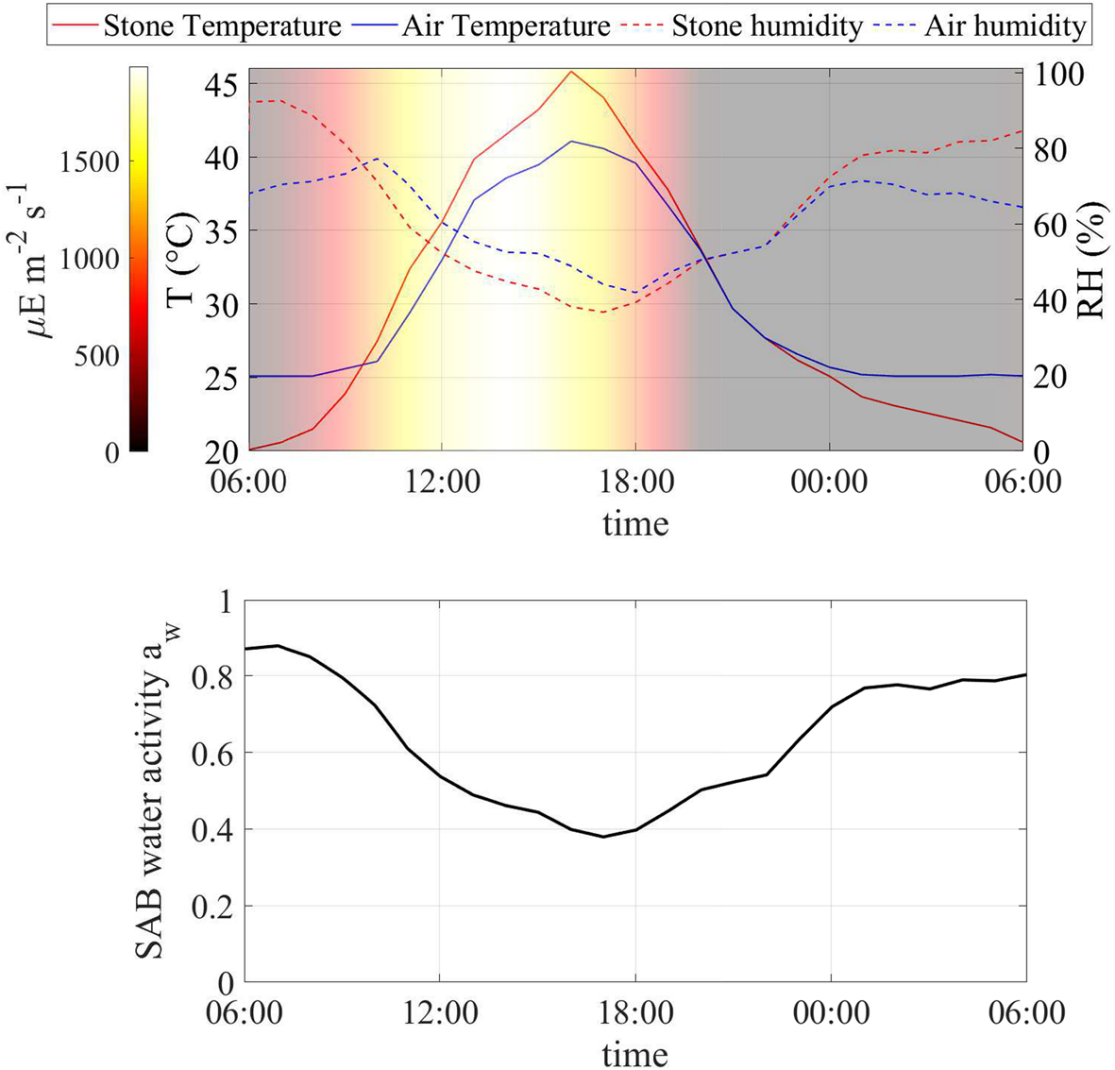
Numerical study 1—(top) Daily profile of air and stone temperatures and relative humidities, and light intensity, on a summer day (June 18–19, 2016) at the Thomas Jefferson Memorial, Washington, DC (USA). (Bottom) Computed SAB water activity (at SAB thickness $\lambda = 15\,\upmu$m). (Red solid) stone temperature, (blue solid) ambient air temperature away from the stone surface, (red dash) relative humidity at stone surface, (blue dash) relative humidity in ambient air away from the stone surface, (black solid) computed water activity. Background colors indicate light intensity ($\upmu$E m$^{-2}$ s$^{-1}$) (Color figure online)

**Fig. 5:**
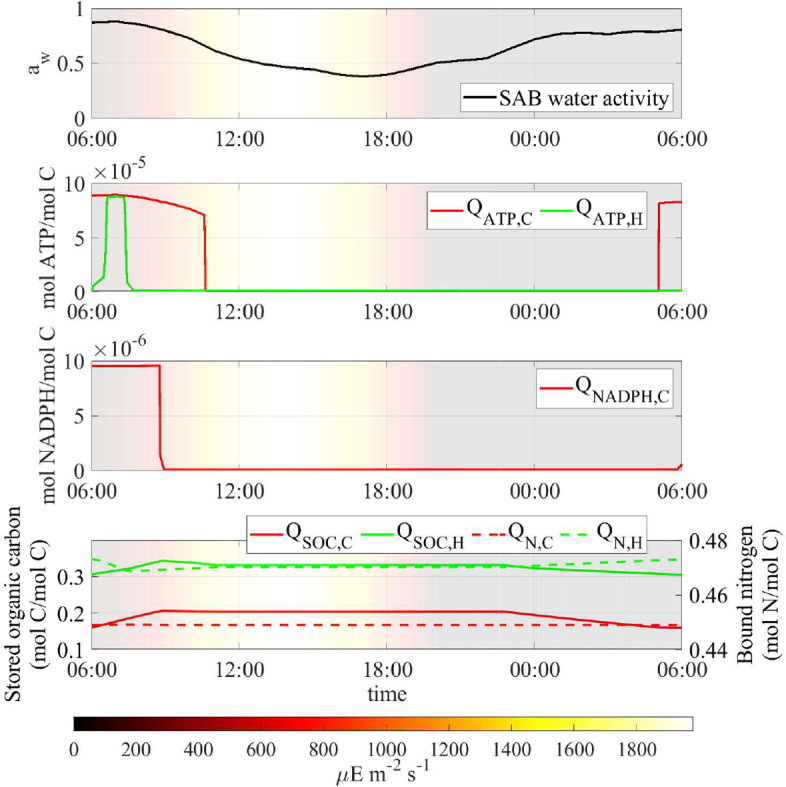
Numerical study 1—computed periodic steady-state profiles of, from top to bottom, SAB water activity, quota of ATP $Q_{ATP,i}$, NADPH $Q_{NADPH,C}$, stored organic carbon $Q_{SOC,i}$ and bound nitrogen $Q_{N,i}$. Background colors indicate light intensity ($\upmu$E m$^{-2}$ s$^{-1}$) (Color figure online)

**Fig. 6:**
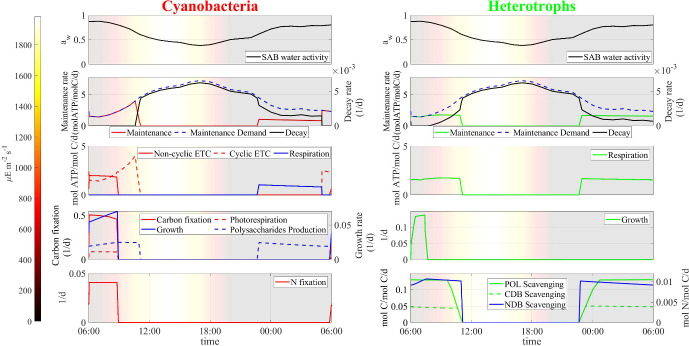
Numerical study 1—computed periodic steady-state profiles of SAB water activity, and main metabolic rates in cyanobacteria (left) and heterotrophs (right). Background colors indicate light intensity ($\upmu$E m$^{-2}$ s$^{-1}$). Note that maintenance demands rise during daylight hours, while most metabolic activity occurs during early morning. The profiles of water activity reported on top—left and right are identical, and serves for a point-by-point comparison with the underlying subplots (Color figure online)

**Fig. 7:**
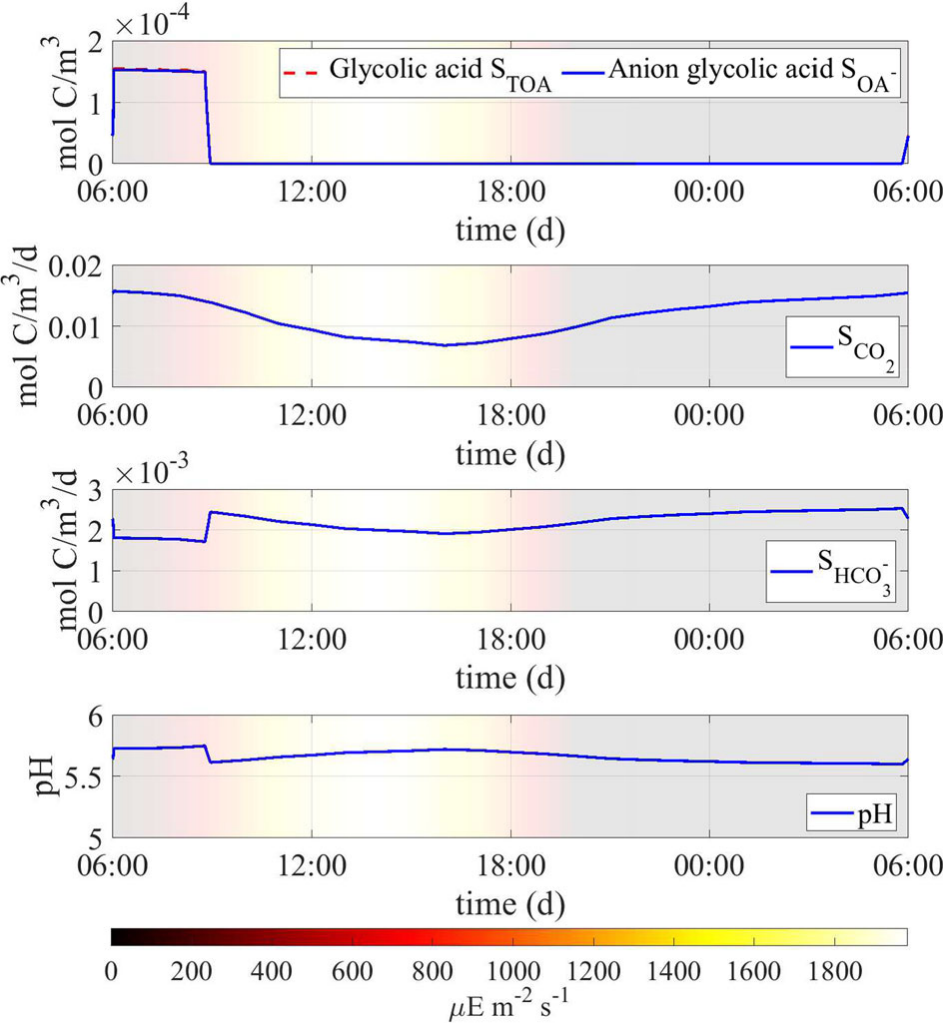
Numerical study 1—computed periodic steady-state profiles of dissolved compounds (total and anion glycolic acid, carbon dioxide and bicarbonate), and pH level. Background colors indicate light intensity ($\upmu$E m$^{-2}$ s$^{-1}$) (Color figure online)

**Fig. 8:**
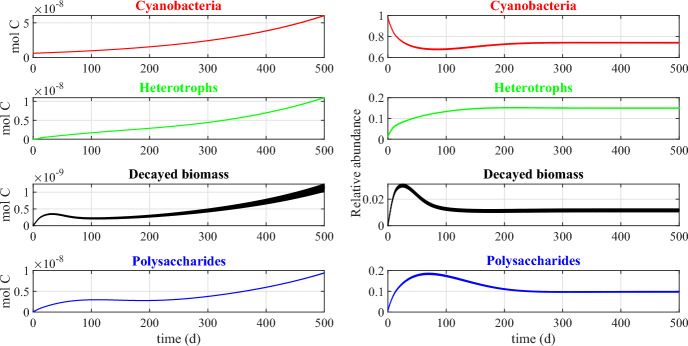
Numerical study 1—time evolution of total mass (left) and relative abundance (right) of SAB solid-phase components: cyanobacteria ($M_C (1+Q_{SOC,C})$), heterotrophs ($M_H (1+Q_{SOC,H})$), decayed biomass ($M_{CDB}$), polysaccharides ($M_{POL}$). Note the different scales on the vertical axes. The broadening in some variable trends is actually due to daily variations that are not clearly discernible on the larger time scale reported in the figure (Color Figure Online)

The first case is based on the environmental profile for a specific, representative summer day at the Thomas Jefferson Memorial. Environmental input variables are illustrated in Fig. 4 (top). As a consequence of radiative heating and cooling effects, stone temperature is lower than the ambient-air temperature under conditions of minimal or absent light, while it is higher during diurnal hours coinciding with solar illumination. This phenomenon has an inverse effect on relative humidity trends, causing the relative humidity at the stone surface to drop below ambient air relative humidity as the temperature increases, and vice versa with temperature decrease. Note that temperature and humidity trends significantly impact SAB activity through water activity, as computed in Fig. 4 (bottom). On this particular day, water activity reaches approximately 0.8 during the early morning and nighttime hours, gradually declining to around 0.4 during the warmest hours of the day. We used the data for this summer day, repeated periodically over many days, to simulate until a periodic state was attained approximately. Note that the periodically extended environmental data has a discontinuity each day at 6AM (because the actual environmental data is not exactly 24-hour periodic), but we do not expect this to significantly impact results, as the discontinuity is effectively smoothed by integration.

After a transient initial phase, computations indicate that the cellular quotas of carbon, nitrogen, ATP, and NADPH, the concentration of soluble compounds and, consequently, the rates of metabolic processes, reach, approximately, periodic profiles, with diel cyclic patterns. Results for the final day of the multi-day simulation are shown in Figs. 5, 6 and 7. The profiles of cellular quotas are illustrated in Figs. 5 and 6 shows the diurnal patterns of the metabolic processes. The model predicts two daily activity periods. First, in the early morning hours, water activity is at its highest and photons are available so photosynthesis and biosynthesis govern the dynamics within the ecosystem. Also, in the early evening water activity is again high enough to allow the production of energy through respiration and the processes of nutrient supply. During the early morning hours, high water activity levels give rise to diminished maintenance requirements, and support energy and NADPH production, via non-cyclic and cyclic electron transport pathways, in sequence, in cyanobacteria, and respiration in heterotrophs. First, when water activity has not yet sharply decreased, photosystem II can work properly and the non-cyclic pathway leads to the production of NADPH which is used, in addition to ATP, for fixing inorganic carbon and nitrogen, and biosynthesizing new cells. At the same time, photorespiration in cyanobacteria leads to the release of organic acids.

Later, though, as light intensity and temperature increases and water activity decreases, the model predicts that the non-cyclic pathway stops. As a result, NADPH concentration in cyanobacteria approaches zero, leading to the cessation of biosynthesis and nitrogen fixation processes. Instead, the cyclic pathway intensifies, leading to the production of ATP that still meets the maintenance demand in cyanobacteria until water activity drops too low. Eventually, water activity declines to values below 0.6 during the midday period, leading to the suppression of all metabolic processes, in agreement with experimental evidences (Wierzchos et al. 2012). The increasing maintenance requirements are no longer met, and cellular decay occurs.

As the sun sets, temperatures decrease and humidity levels rise, resulting in a subsequent increase in water activity that triggers the second daily activity period. This shift reactivates metabolic processes: respiration of stored carbon results in the production of new energy that fully or partially covers maintenance costs. Biochemical processes for nutrient acquisition, including scavenging of polysaccharides and decayed biomass by heterotrophs, as well as nitrogen fixation by cyanobacteria, occur in the model during both the early morning and after sunset, under favorable water activity conditions.

It’s worth mentioning that, even during periods of the day when the water activity level allows functioning of the SAB, it can still impose a significant limitation on metabolic rates. As a consequence, the cellular quotas of carbon and nitrogen experience only slight variations throughout the entire day, see Fig. 5, bottom. In particular, even when ATP and NADPH are not limiting factors, growth and respiration processes in both cyanobacteria and heterotrophs are notably slow due to the low stored carbon content within the cells, while nitrogen levels are not found to be limiting.

Model microbial dynamics are strongly influenced by the pH level in the SAB, which, in turn, is affected by the concentrations of ionic species, i.e. bicarbonate and organic acids. From this perspective, heterotrophic bacteria have the potential to play a significant role in pH regulation through their capacity to degrade non-volatile organic acids. Figure 7 provides model predictions for the daily profiles of concentrations of dissolved ionized compounds, and their effects on pH. Organic acid production occurs during the early morning when optimal water and light conditions for photosynthesis and photorespiration processes occur. However, at the same time, rapid absorption of acids by heterotrophs keeps acid concentration within the SAB at low values. Accordingly, in the presence of heterotrophs, non-volatile organic acids play a marginal role in the pH level of the ecosystem; rather, pH slightly increases in the early morning due to the consumption of bicarbonate during photosynthesis. Note also that, throughout the rest of the day, pH exhibits very moderate oscillations around mildly acidic levels mostly due to temperature variations: CO$_2$ is less soluble at higher temperatures, resulting in a lower concentration of bicarbonate anions and a consequent higher pH level. See Sect. 4.2.2 for a study of pH levels when heterotrophs are not present.

Finally, Fig. 8 shows the dynamics over time of the masses of cyanobacteria, heterotrophs, and polysaccharides, and of decayed biomass (left), and their respective relative abundances in the SAB (right). Note that the model does not include any decay mechanism other than through inadequate maintenance. Thus, once the system has reached a periodic state, i.e. once the cellular quotas, the substrate concentrations and the metabolic rates has achieved the aforementioned periodic profiles, either SAB extinction or constant daily SAB growth is to be expected. Which possibility occurs depends on the daily environmental conditions. Since the SAB thickness and density are constant (in the model), this corresponds here to a slow expansion of both SAB area coverage and SAB volume over time, indicating perhaps the colonization of new stone surfaces, as is consistent with observations. The ultimate limit on total biomass is not considered here, though.

**Fig. 9:**
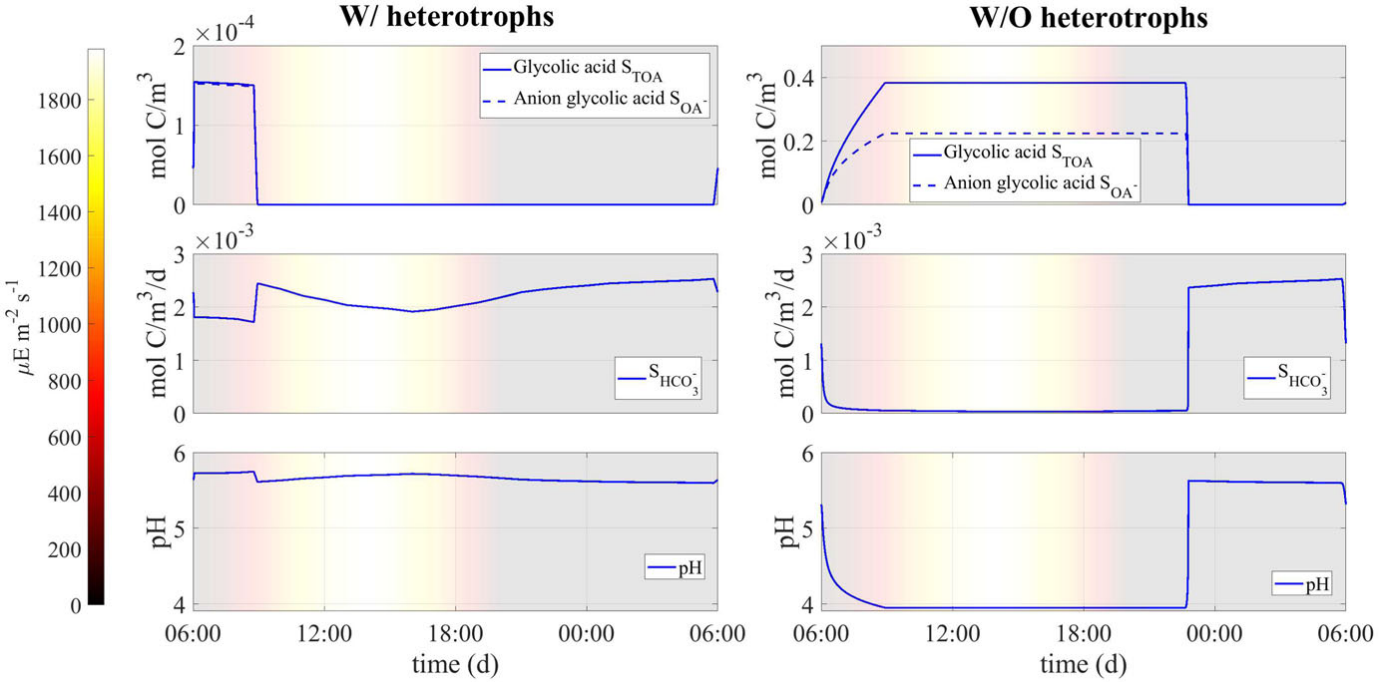
Numerical study 2—computed periodic steady-state profiles of total and anion glycolic acid, bicarbonate, and pH level in the presence (left) and absence (right) of heterotrophs. Background colors indicate light intensity ($\upmu$E m$^{-2}$ s$^{-1}$). Note the different scales on the vertical axes (Color figure online)

**Fig. 10:**
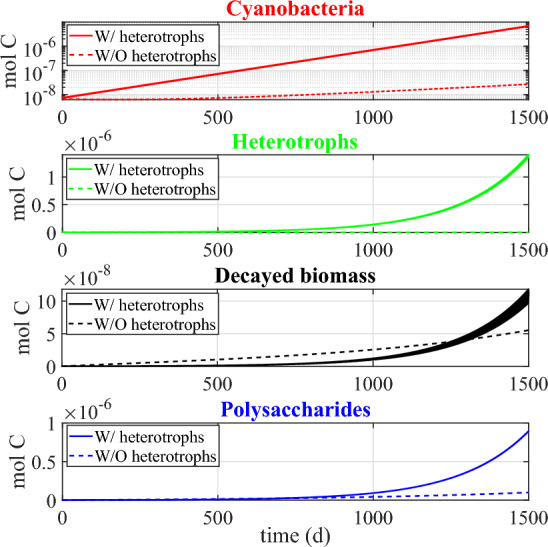
Numerical study 2—time evolution of mass of SAB solid-phase components in the presence (solid lines) and absence (dashed lines) of heterotrophs: cyanobacteria ($M_C (1+Q_{SOC,C})$), heterotrophs ($M_H (1+Q_{SOC,H})$), polysaccharides ($M_{POL}$), decayed biomass ($M_{CDB}$). The mass of cyanobacteria is presented on a logarithmic scale to enhance visualization. The broadening in some variable trends is actually due to daily variations that are not clearly discernible on the larger time scale reported in the figure (Color Figure Online)

**Fig. 11:**
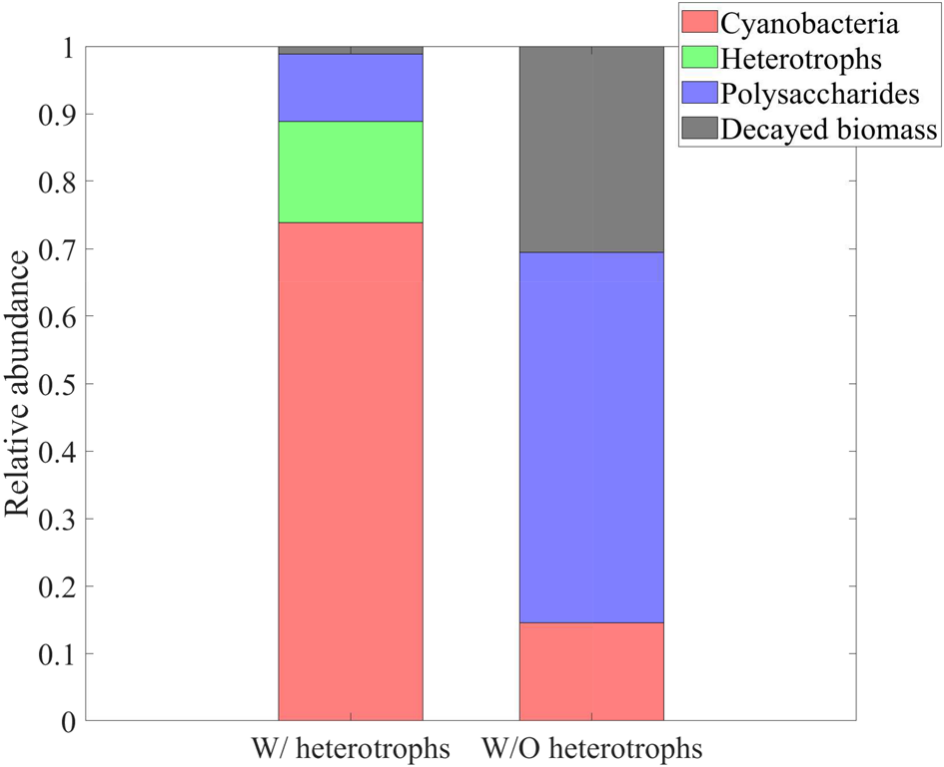
Numerical study 2—approximate steady-state SAB composition in the presence (left) and absence (right) of heterotrophs. SAB components: cyanobacteria ($M_C (1+Q_{SOC,C})$), heterotrophs ($M_H (1+Q_{SOC,H})$), polysaccharides ($M_{POL}$), decayed biomass ($M_{CDB}$) (Color Figure Online)

**Fig. 12:**
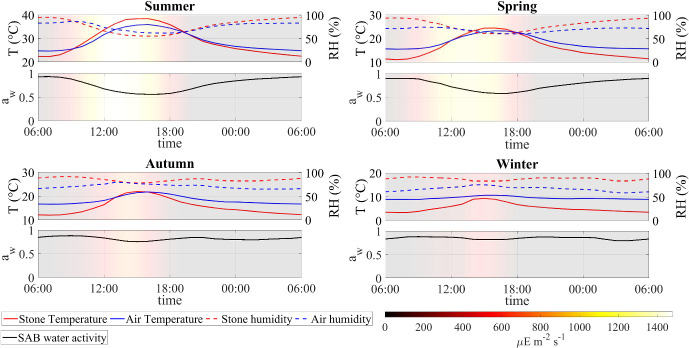
Numerical study 3—daily profiles of air and stone temperatures, relative humidities, light intensity and computed SAB water activity (at SAB thickness $\lambda =15\,\upmu$m), averaged by season, at the Thomas Jefferson Memorial, Washington, DC (USA). (Red solid) stone temperature, (blue solid) ambient air temperature away from the stone surface, (red dash) relative humidity at stone surface, (blue dash) relative humidity in ambient air away from the stone surface, (black solid) computed water activity. Background colors indicate light intensity ($\upmu$E m$^{-2}$ s$^{-1}$). Note the same temperature range length ($20\,^\circ$C) on the left y-axis (Color figure online)

**Fig. 13:**
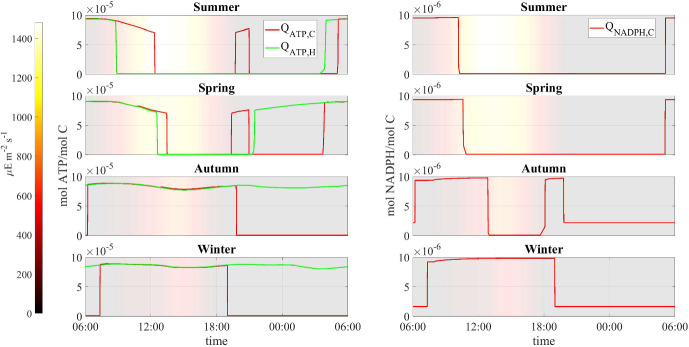
Numerical study 3—computed periodic profiles of the quota of ATP $Q_{ATP,i}$ and NADPH $Q_{NADPH,C}$, season by season. Background colors indicate light intensity ($\upmu$E m$^{-2}$ s$^{-1}$) (Color figure online)

**Fig. 14:**
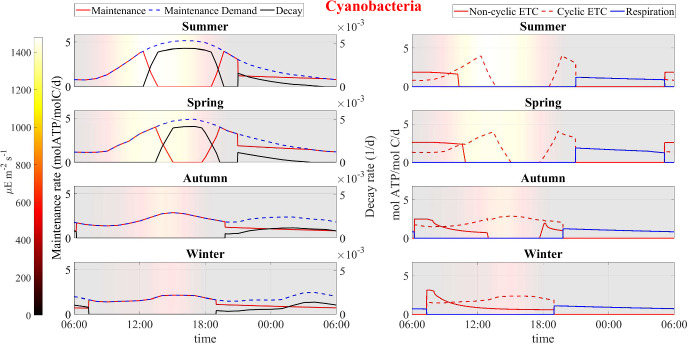
Numerical study 3—computed periodic profiles of maintenance, decay and ATP production rates in cyanobacteria, season by season. Background colors indicate light intensity ($\upmu$E m$^{-2}$ s$^{-1}$) (Color figure online)

**Fig. 15:**
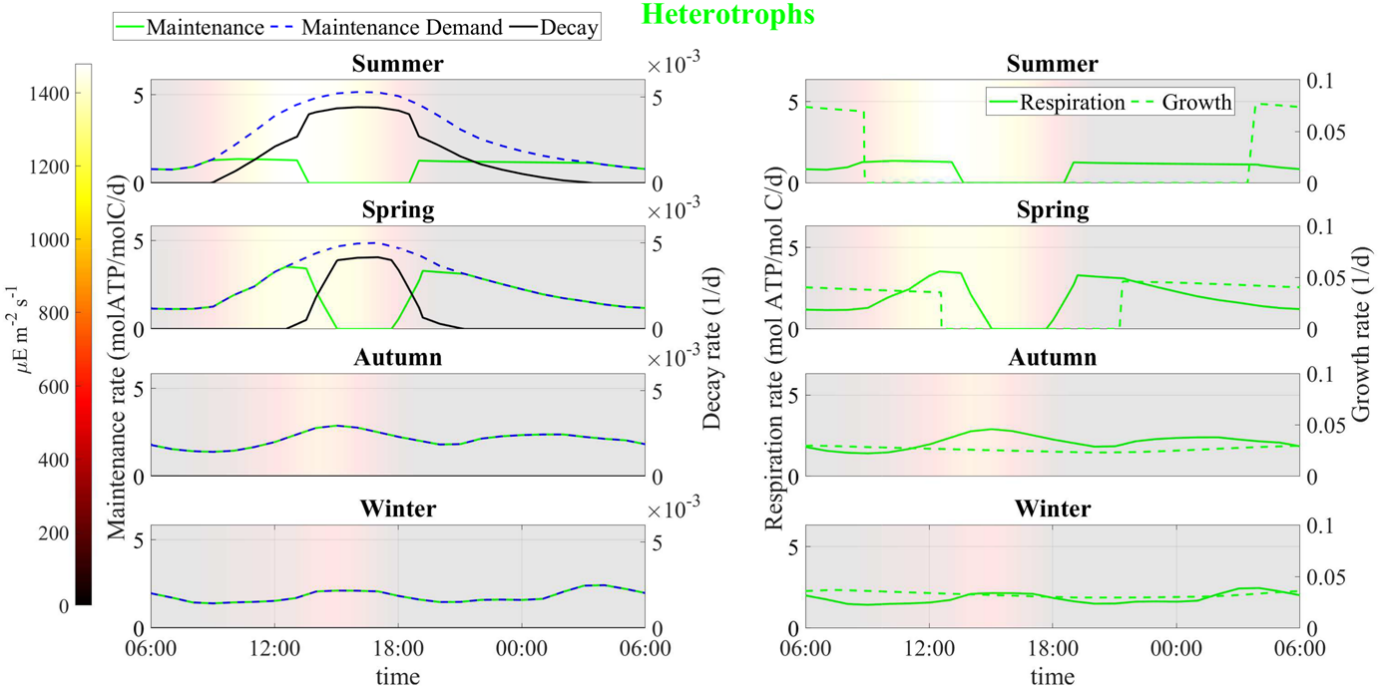
Numerical study 3—computed periodic profiles of main metabolic rates in heterotrophs, season by season. Background colors indicate light intensity ($\upmu$E m$^{-2}$ s$^{-1}$) (Color figure online)

**Fig. 16:**
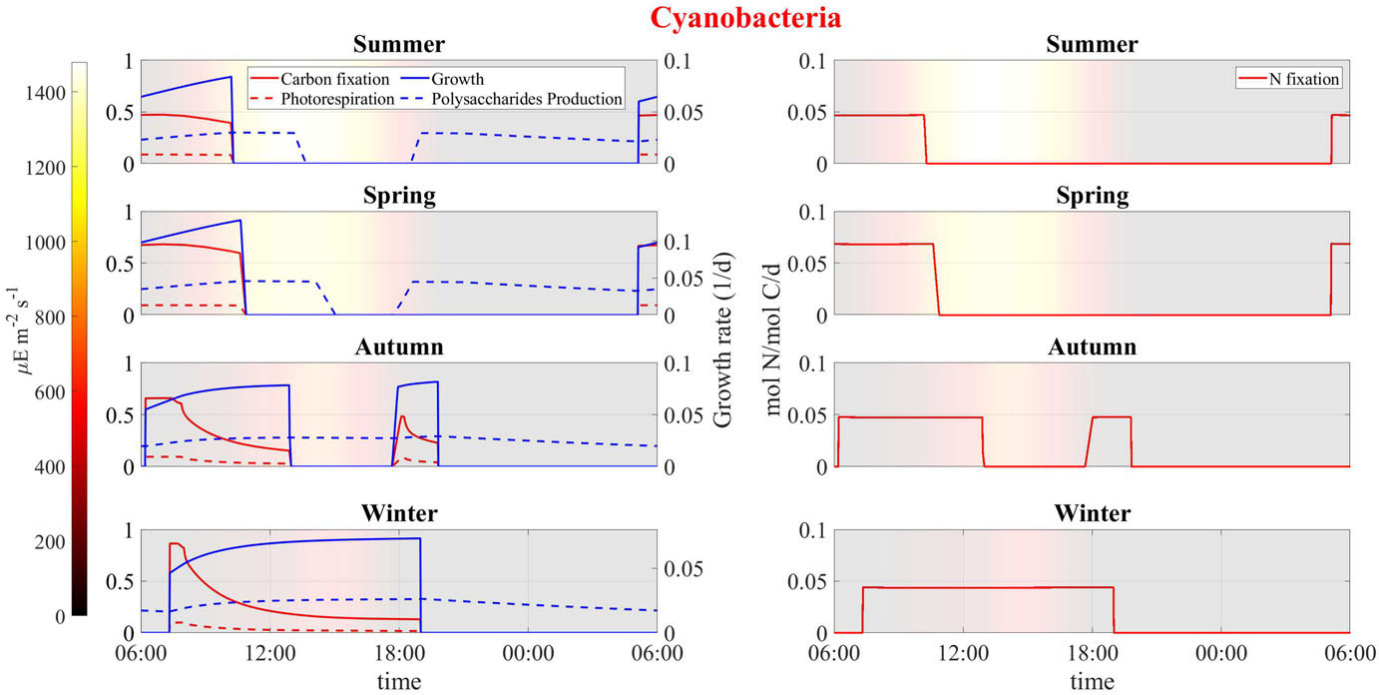
Numerical study 3—computed periodic profiles of photorespiration, carbon fixation, growth, polysaccharide production and nitrogen fixation rates in cyanobacteria, season by season. Background colors indicate light intensity ($\upmu$E m$^{-2}$ s$^{-1}$) (Color figure online)

In contrast to total SAB biomass, a pseudo-stationary relative composition is achieved by the SAB as the model reaches its approximate periodic state. Note that the long transient phase of the biofilm composition is a result of our arbitrary initial conditions. Cyanobacteria make up the dominant component of the SAB, although substantial amounts of heterotrophs, polysaccharides and decayed biomass are also present. As an aside, we mention that it is difficult to quantitatively compare the mathematical model predictions reported in Fig. 8 to 16S rRNA gene sequencing data in Fig. 1. While 16S rRNA gene sequencing provides information about 16S rRNA in a sample, the model instead tracks total biological mass. Conversions between sequencing data and total biomass introduces unknowns due to the dynamic nature of bacterial communities and their responses to environmental factors, among other things. As one example, the 16S rRNA gene sequencing was based on RNA thus providing data about active bacteria present at a given instant, information which can vary significantly in time as a function of multiple environmental variables.

Remark: we conducted an additional numerical study to model the SAB dynamics in a different environment, the marble roof of Federal Hall National Memorial in New York City. Unlike the Thomas Jefferson Memorial, this marble roof is located in an urban canyon that affects its environmental conditions. Following the same approach used in numerical study 1 above, we periodically extended the environmental profile on a specific, representative summer day at Federal Hall National Memorial, extracted from the data gathering campaign reported in Tenore et al. (2023a). Results have been provided in the Supplementary Material, but the metabolic patterns are similar to those observed in numerical study 1 above of the Thomas Jefferson Memorial environment. This suggests a certain level of robustness of SAB systems.

#### Numerical study 2: Role of the heterotrophs in the SAB ecosystem

To elucidate the interactions between heterotrophs and cyanobacteria in SABs, we performed two simulations: one in the presence (initial conditions $M_{H,0}$=0.008 $M_{0}$) and one in the absence (initial conditions $M_{H,0}$=0) of heterotrophs. The rest of the initial and environmental conditions are set to be the same as in numerical study 1. Results are summarized in Figs. 9, 10 and 11.

Figure 9 shows the daily profiles of the concentrations of bicarbonate and organic acids, and their impacts on the pH level, in the presence and absence of heterotrophs. From this figure, it is clear that the model predicts that the presence of heterotrophs strongly influences the concentration of organic acids and pH levels. In the early morning hours, cyanobacteria exhibit intense photosynthetic activity, releasing organic acids as they uptake bicarbonate, thus maintaining charge equilibrium within their cells. When heterotrophs are present in the ecosystem, the organic acids released by cyanobacteria are immediately consumed. As a result, the concentration of acids in the ecosystem remains nearly zero for most of the day and increases only slightly during the early morning. For this reason, the pH level remains relatively constant, and small fluctuations are attributed, as explained in the previous section, to changes in the bicarbonate concentration and CO$_2$ solubility associated with temperature variations. When heterotrophs are not present in the ecosystem, organic acids released during the morning accumulate in the SAB, reaching a significant concentration. They are reabsorbed by cyanobacteria only at the end of the daytime, to balance the charge variation due to the release of bicarbonate from the cell to the medium during respiration. This affects the pH, which decreases and remains approximately 4 for most of the day. The absence of heterotrophs also indirectly affects the availability of bicarbonate for cyanobacteria. As the pKa of the CO$_2$-HCO$_3$ equilibrium reaction is approximately 6.36, lower pH values correspond to a lower concentration of bicarbonate in the ecosystem, as shown in the Fig. 9.

These outcomes may play a fundamental role in the metabolic processes of microorganisms and influence the composition of the SAB ecosystem, as can be seen in Figs. 10 and 11. In particular, the model predicts that the lower pH level and reduced bicarbonate concentration in the ecosystem without heterotrophs limit the biosynthesis processes of cyanobacteria. The result is a reduction in cyanobacterial biomass and overall ecosystem growth, along with a higher relative abundance of polysaccharides and decayed biomass, which accumulate more in the absence of the heterotrophic consumption. From this perspective, potential alternative mechanisms may be necessary to preserve the resilience and viability of SAB ecosystems in the absence of heterotrophs.

**Fig. 17:**
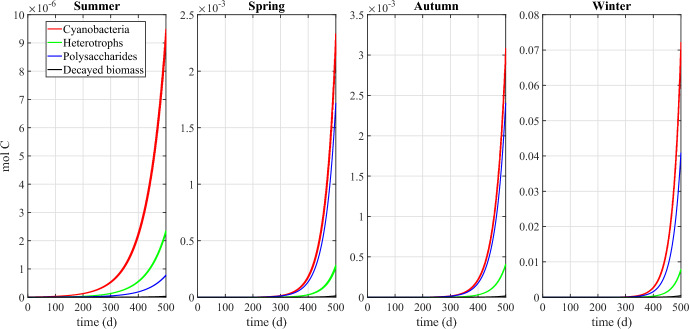
Numerical study 3—time evolution of mass of SAB solid-phase components, season by season: cyanobacteria ($M_C (1+Q_{SOC,C})$), heterotrophs ($M_H (1+Q_{SOC,H})$), polysaccharides ($M_{POL}$), decayed biomass ($M_{CDB}$). Note the different scales on the vertical axes (Color Figure Online)

**Fig. 18:**
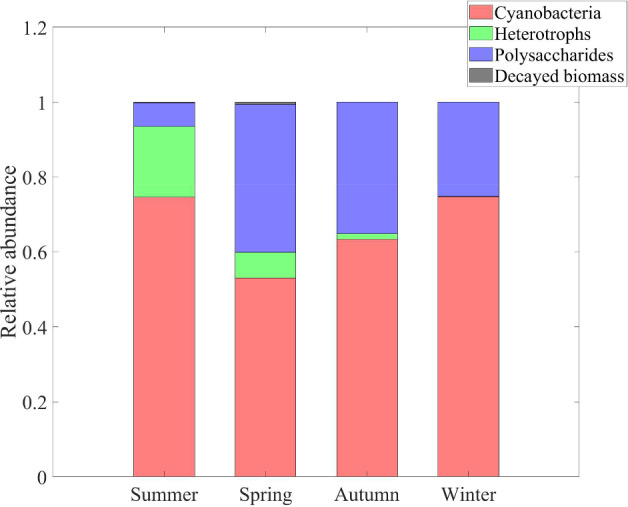
Numerical study 3—approximate steady-state SAB composition, season by season. SAB components: cyanobacteria ($M_C (1+Q_{SOC,C})$), heterotrophs ($M_H (1+Q_{SOC,H})$), polysaccharides ($M_{POL}$), decayed biomass ($M_{CDB}$) (Color Figure Online)

#### Numerical study 3: SAB dynamics across four seasons

Here we compare model results for different seasons using seasonally averaged environmental profiles at the Thomas Jefferson Memorial: Spring (March-May), Summer (June-August), Autumn (September-November), and Winter (December-February). That is, an averaged “day” is constructed for each of the four seasons by averaging data over the entirety of that season. These seasonally averaged profiles are repeated periodically day by day throughout the simulation time (one simulation for each season). Initial values are the same as for the previous numerical studies. Numerical results are showed in Figs. 12, 13, 14, 15, 16, 17 and 18 and refer to the last day of simulation, when the cellular quotas of carbon, nitrogen, ATP, and NADPH have reached an approximately periodic pattern.

Figure 12 depicts the daily profiles of temperature, humidity, and water activity conditions for the distinct seasons. Notably, light intensity is lower during colder seasons, resulting in the stone maintaining a lower temperature throughout the day compared to the air. Seasonal differences in temperature and humidity affect the SAB water activity profile: during summer and spring, significant fluctuations in water activity are observed, occurring only to a lesser extent during winter and autumn when water activity remains relatively high throughout the day. Overall, there is an increase in water activity from the warmest season to the coldest season.

Figure 13 displays the daily ATP and NADPH profiles. In accordance with the water activity conditions, the model predicts that during summer and spring, cellular quotas of ATP and NADPH are high at localized times of the day when water activity is optimal while they remain high for most of the day during winter and autumn. These results can also be explained by analyzing Figs. 14 and 15 that show the daily kinetics of maintenance, decay, and energy production mechanisms carried out by cyanobacteria and heterotrophs, respectively. During summer and spring, cells face challenges in carrying out their metabolic activities during the warmest hours of the day. Specifically, limited water availability and high osmotic tension impose two detrimental effects on microorganisms: high maintenance costs and inhibition of ATP-production processes of photosynthesis and respiration. These conditions lead to a state of decay. Conversely, during autumn and winter, water activity levels remain elevated. As a result, maintenance demand is lower, and the processes of photosynthesis and respiration are carried out efficiently. Under these circumstances, ATP production covers maintenance costs throughout the day, or nearly so, thereby sustaining consistently high cellular ATP quotas. Note though that the model does not include direct temperature effects on metabolic activity; we assume in part for simplicity that the specialized SAB community has developed a metabolic stress response to temperature fluctuations (Villa and Cappitelli 2019).

Similar trends are observed in predictions for carbon fixation processes, biosynthesis of new cells and polysaccharide production (see Fig. 16). During spring and summer, both cyanobacteria and heterotrophs are found to grow only in the early morning, when optimal light and high water activity conditions occurs. During autumn, cyanobacteria biosynthesis processes also occur in the late hours of the day, being inhibited only in the middle of the day, while in winter, cyanobacteria grow continuously during the daytime. During both autumn and winter, heterotrophic bacteria grow throughout the day, albeit at a reduced rate when compared to the warmer seasons owing to diminished biomass decay and consequent lower availability of organic carbon and nitrogen.

Figures 17 and 18 illustrate the growth patterns of SAB constituents and the pseudo steady-state SAB composition, season by season. As we previously observed, the seasonal fluctuations in water activity play a pivotal role in shaping these growth trends. Water activity increases from the warmest to the coldest season, and this is also reflected in SAB growth. In summer, the growth of all components is minimal, while it becomes maximal during winter. Furthermore, in colder seasons, cyanobacteria grow at a faster rate than heterotrophs. This can be attributed to the higher water activity that enhances cyanobacterial growth while limiting decay, leaving only organic acids as a carbon source. The result is reduced amounts of organic carbon and nitrogen available for heterotrophs. Accordingly, an increase in the relative abundance of cyanobacteria and a decline in that of heterotrophs are observed from the warmest season to the coldest one.

## Discussion and conclusions

Environmental SABs provide examples, albeit somewhat extreme, of the importance of environmental conditions for microbial communities that are exposed to them. ‘Weather’ and its variability are not always included in the list of stresses that microbial communities suffer, generally, but they are often present, and in fact most microorganisms are subject to them (and have been over the history of life on earth, and likely also over the history of extraterrestrial life in the solar system, if it is present). It is thus a useful exercise to attempt to describe and model the interactions between environmental microbial communities, here SABs, and their environments.

The proposed model offers a detailed representation of the interactions between cyanobacteria and heterotrophic bacteria within subaerial biofilms (SAB). Its innovation lies in integrating key environmental factors, such as daily cycles of temperature, humidity, and light intensity, which crucially affect microbial activity through their impact on water availability and metabolic kinetics. Furthermore, the model simulates real-world scenarios, specifically the marble roofs of the Thomas Jefferson Memorial and Federal Hall National Memorial, revealing distinct daily activity periods and long-term survivability conditions, thereby providing a deeper understanding of SAB ecology. The aim is to characterize SABs and their environments as closely coupled systems.

Numerical investigation points to the impact of environmental conditions, weather essentially, on stone SAB ecosystems. Their fluctuations significantly influence dynamics within SABs. On longer, seasonal time scales, temperature and relative humidity changes are more pronounced during the warmer seasons (summer and spring), resulting in more substantial variations in SAB water activity. Conversely, typical autumn and winter days exhibit more moderate environmental variability, sustaining higher water activity levels throughout the day. These variations have a direct impact on microbial metabolic pathways, highlighting the importance of considering environmental variability when assessing SAB dynamics, as outlined in Prieto et al. (2020).

On a 24 h time scale, we observe that high water activity levels in the early morning hours support enhanced metabolic activity, including photosynthesis, production of ATP and NADPH, and biomass synthesis. However, water activity declines during the day due to increased temperatures and reduced relative humidities, imposing challenges on microorganisms, leading to limited metabolic activity, higher maintenance costs and subsequent decay. This observed daily pattern aligns with the findings in the experimental literature (Mazor et al. 1996), suggesting the central role of water activity in facilitating metabolic processes. Organic acids, decayed biomass and polysaccharides secreted by cyanobacteria lead to availability of recyclable organic nutrients for heterotrophic metabolic activities. On colder days, sustained water availability promotes more continuous metabolic activity in both cyanobacteria and heterotrophs throughout the day, with reduced maintenance costs and improved energy production. As a result, slower decay of active species and polysaccharides production occur, leading to lower availability of nutrients in the ecosystem; heterotrophs still grow consistently using organic acids, albeit at a slower rate compared to the warmer months. The seasonal variations in metabolic activity suggest the need for adaptability of SABs to changing environmental conditions, as discussed in Villa et al. (2015). We note that this issue is not explored here.

The model also predicts consequences of basic SAB community ecology. Results in the presence and absence of heterotrophs elucidate the ecological role of heterotrophs within SABs. The role of photorespiration in cyanobacteria emerges as an important mechanism in the release of organic acids, decreasing pH levels within the ecosystem. Notably, such acids are generally non-volatile, so they remain in the system. However, the model suggests that the presence of heterotrophs alleviates this impact: organic acids produced by cyanobacteria are rapidly consumed by heterotrophs, maintaining low acid concentrations and relatively stable pH levels in the SAB. In contrast, in the absence of heterotrophs, organic acids accumulate in the SAB during the early morning hours of intense metabolic activity and are only reabsorbed by cyanobacteria in the late hours of the day, during respiration when photosynthesis is not occurring. This process results in a significantly low pH level for most of the day and influences the evolution and composition of the SAB ecosystem: the reduced pH level and bicarbonate concentration limit photosynthetic activity of cyanobacteria. Overall, both the total amount and the relative abundance of cyanobacteria are predicted to be higher in the presence of the heterotrophic component, in agreement with what is observed experimentally in Villa et al. (2015). These results suggest a broader conclusion: any non-volatile byproduct of SABs builds up within the ecosystem and may impact the metabolic activity of its inhabitants. From this perspective, the presence of diverse microbial species with specific roles and functions can assist in removing harmful waste products and contribute to the biodiversity and balance of the ecosystem.

It is important to note here that we consider SAB thicknesses on the order of 10–20 microns, inhabited by cyanobacteria and heterotrophs, in accordance with our field observations at the particular sites we model. The thinness of this layer allows us to neglect the spatial variability of the SAB and consider the biofilm as a fully penetrated domain, assuming a steady-state formulation for volatile compound solubilization, and water evaporation/condensation processes. These hypotheses may be well-met in many subaerial environments which experience significant fluctuations in water availability and periods of water stress. In such environments, water activity emerges as the limiting factor for growth, and the thickness becomes an intrinsic characteristic of the microbial aggregate, as noted in Tenore et al. (2023a). However, locally higher water and nutrient availability may support the formation of macroscopic aggregates, characterized by variable thickness and taxonomy. Also, some laboratory-cultivated SABs (Villa et al. 2015) or SABs growing in very humid environments (Ramírez et al. 2010) can reach thicknesses of 50–100 microns or more, leading to diffusive transport limitations.

In this study, we track pH level assuming the liquid water volume to be the same as the SAB volume, neglecting possible local variations in liquid water, which could affect the processes of solubilization and precipitation of dissolved compounds. Furthermore, we have neglected chemical interactions with the marble substratum, a reasonable simplification for pH values exceeding 4.5, beyond which the physico-chemical processes associated with marble corrosion are considered negligible (Guidobaldi and Mecchi 1993). In our simulations, the pH level consistently remains above that threshold, except when heterotrophs are absent. In the latter case, the pH level drops to approximately 4 for a portion of the day, a level that is considered moderately concerning for marble corrosion (Guidobaldi and Mecchi 1993). Accordingly, the model proposes an intriguing hypothesis: heterotrophs potentially play a protective role against marble corrosion.

Another caveat to be mentioned: water activity is here estimated at steady-state conditions, under the assumption that the timescales of evaporation and condensation processes are short in comparison to those of biological processes. Notably, though, experimental evidence suggests that EPS is capable of retaining water and affecting the rate of water exchange between the SAB and the atmosphere (Zammit et al. 2011). In this regard, some attention to transient water dynamics in the SAB-stone system, as well as the role of EPS in the process  may be warranted. Relatedly, perhaps, we regard here the stone substratum as a passive and non-permeable layer. It may be the case, though, that it can transiently retain water and so serve as a temporary SAB water source.

## Supplementary Information

Below is the link to the electronic supplementary material.

Supplementary file 1 (pdf 1710 KB)

## Data Availability

The data that has been used is confidential.

## Detailed mathematical framework

Together with the initial conditions, the model is completely described by the following set of ODEs (parameters are discussed in Appendix B):

44 $$\begin{aligned} &  \!\!\!\!\frac{dM_C}{dt} = r_{C,growth}-r_{C,d}, \ M_C(0)=M_{C,0},\nonumber \\ &  \quad \frac{dQ_{SOC,C}}{dt} = \frac{r_{fix}}{M_C} - \frac{r_{C,growth}}{M_C} - \frac{r_{C,resp,}}{M_C} - \frac{r_{POL \ prod}}{M_C} \nonumber \\ &  \quad + \frac{r_{C,TOA \ uptake}}{M_C} -r_{C,d} Q_{SOC,C}, \end{aligned}$$

45 $$\begin{aligned} &  Q_{SOC,C}(0)=Q_{SOC,C,0},\nonumber \\ &  \quad \frac{dQ_{ATP,C}}{dt} = -3 \ \frac{r_{fix}}{M_C} - 0.77 \ \frac{r_{C,growth}}{M_C} + \frac{38}{6} \ \frac{r_{C,resp}}{M_C} - \frac{r_{photo}}{M_C} + 3 \ \frac{r_{nc}}{M_C} \end{aligned}$$

46 $$\begin{aligned} &  \quad + \frac{r_{cy}}{M_C} - 8 \ \frac{r_{Nfix}}{M_C} - \frac{r_{C,main}}{M_C} -r_{C,d} Q_{ATP,C}, \ Q_{ATP,C}(0)=Q_{ATP,C,0},\nonumber \\ &  \quad \frac{dQ_{NADPH,C}}{dt} = -2 \ \frac{r_{fix}}{M_C} - 0.05 \ \frac{r_{C,growth}}{M_C} - 2.5 \ \frac{r_{photo}}{M_C} \nonumber \\ &  \quad + 2 \ \frac{r_{nc}}{M_C} - 2 \ \frac{r_{Nfix}}{M_C} \end{aligned}$$

47 $$\begin{aligned} &  \quad -r_{C,d} Q_{NADPH,C}, \ Q_{NADPH,C}(0)=Q_{NADPH,C,0}, \end{aligned}$$

48 $$\begin{aligned} &  \frac{dQ_{N,C}}{dt} = -0.2 \ \frac{r_{C,growth}}{M_C} + \frac{r_{Nfix}}{M_C} -r_{C,d} Q_{N,C}, \ Q_{N,C}(0)=Q_{N,C,0}, \end{aligned}$$

49 $$\begin{aligned} &  \frac{dM_H}{dt} = r_{H,growth}-r_{H,d}, \ M_H(0)=M_{H,0},\nonumber \\ &  \quad \frac{dQ_{SOC,H}}{dt} = - \frac{r_{H,growth}}{M_H} - \frac{r_{H,resp}}{M_H}\nonumber \\ &  \quad + \frac{r_{H,TOA \ uptake}}{M_H} + \frac{r_{POL \ scav}}{M_H}+ \frac{r_{CDB \ scav}}{M_H} \end{aligned}$$

50 $$\begin{aligned} &  \quad -r_{H,d} Q_{DOC,H}, \ Q_{SOC,H}(0)=Q_{SOC,H,0},\nonumber \\ &  \quad \frac{dQ_{ATP,H}}{dt} = - 0.77 \ \frac{r_{H,growth}}{M_H} + \frac{38}{6} \ \frac{r_{H,resp}}{M_H} - \frac{r_{H,main}}{M_H} -r_{H,d} Q_{ATP,H}, \end{aligned}$$

51 $$\begin{aligned} &  Q_{ATP,H}(0)=Q_{ATP,H,0}, \end{aligned}$$

52 $$\begin{aligned} &  \frac{dQ_{N,H}}{dt} = -0.2 \ \frac{r_{H,growth}}{M_H} + \frac{r_{NDB \ scav}}{M_H} - r_{H,d} Q_{N,H}, \ Q_{N,H}(0)=Q_{N,H,0}, \nonumber \\ \end{aligned}$$

53 $$\begin{aligned} &  \frac{dM_{POL}}{dt} = r_{POL \ prod}-r_{POL \ scav}, \ M_{POL}(0)=M_{POL,0},\nonumber \\ &  \quad \frac{dM_{CDB}}{dt} = r_{C,d} \ (1 + Q_{SOC,C}) \nonumber \\ &  + r_{H,d} \ (1 + Q_{SOC,H}) - r_{CDB \ scav}, \end{aligned}$$

54 $$\begin{aligned} &  M_{CDB}(0)=M_{CDB,0}, \end{aligned}$$

55 $$\begin{aligned} &  \frac{dM_{NDB}}{dt} = r_{C,d} \ (0.2 + Q_{N,C}) \nonumber \\ &  \quad + r_{H,d} \ (0.2 + Q_{N,H}) - r_{NDB \ scav}, \ M_{NDB}(0)=M_{NDB,0}, \end{aligned}$$

56 $$\begin{aligned} &  \frac{dS_{TOA}}{dt} = \frac{r_{photo}}{V} - \frac{r_{C,TOA \ uptake}}{V} - \frac{r_{H,TOA \ uptake}}{V}, \ S_{TOA}(0)=S_{TOA,0}, \end{aligned}$$

57 $$\begin{aligned} &  \frac{dS_{OA^-}}{dt} = - r_{OA^-,eq}, \ S_{OA^-}(0)=S_{OA^-,0}, \end{aligned}$$

58 $$\begin{aligned} &  \frac{dS_{HCO^-_3}}{dt} - \frac{S_{HCO^-_3}}{DIC} \ \frac{r_{fix}}{V} + r_{HCO_3^-,eq}, \ S_{HCO^-_3}(0)=S_{HCO^-_3,0}. \end{aligned}$$

Limiting functions included in the biochemical kinetics (13)–(25) and  (30)–(38) are

$$\begin{aligned} f_{C,nc}= &  \min \{\phi _{ATP,C,2};\phi _{NADPH,2};\phi _{I,1};\phi _{aw,1}\}, \\ f_{C,cy,1}= &  \min \{\phi _{ATP,C,2};\phi _{I,2};\phi _{aw,1}\}, \\ f_{C,cy,2}= &  \min \{\phi _{ATP,C,2};\phi _{I,2};\phi _{aw,2}\}, \\ f_{C,fix}= &  \min \{\phi _{SOC,C,2};\phi _{ATP,C,1};\phi _{NADPH,1};\phi _{I,3};\phi _{DIC};\phi _{pH}\}, \\ f_{C,Nfix}= &  \min \{\phi _{ATP,C,1};\phi _{NADPH,1};\phi _{N,C,2};\phi _{aw,3}\}, \\ f_{C,growth}= &  \min \{\phi _{SOC,C,1};\phi _{ATP,C,1};\phi _{NADPH,1};\phi _{N,C,1};\phi _{I,3}\}, \\ f_{C,pol}= &  \min \{\phi _{SOC,C,1};\phi _{aw,2}\}, \\ f_{C,resp}= &  \min \{\phi _{ATP,C,2};\phi _{SOC,C,1};\phi _{I,4};\phi _{aw,3}\}, \\ f_{C,uptake}= &  \min \{\phi _{SOC,C,2},\phi _{TOA};\phi _{I,4};\phi _{aw,3}\}, \\ f_{C,main}= &  {\left\{ \begin{array}{ll} f^*_{main}, & \text {if}\ Q_{ATP,C}>0 \\ f^*_{main}, & \text {if}\ Q_{ATP,C}=0, \ \omega _C \ge (1-a_w) \ k_{main} \\ \omega _C, & \text {if}\ Q_{ATP,C}=0, \ \omega _C<(1-a_w) \ k_{main} \end{array}\right. }, \ \end{aligned}$$

where

$$\begin{aligned} f^*_{main}= &  k_{main} \ (1-a_w), \\ \omega _C= &  3 \frac{r_{nc}}{M_C}+ \frac{r_{cy}}{M_C} + \frac{38}{6} \frac{r_{C,resp}}{M_C}, \\ f_{C,d}= &  {\left\{ \begin{array}{ll} 0, & \text {if}\ Q_{ATP,C}>0 \\ 0, & \text {if}\ Q_{ATP,C}=0, \ \omega _C \ge (1-a_w) \ k_{main} \\ k_d \ (1-a_w) \ (1-\gamma _C), & \text {if}\ Q_{ATP,C}=0, \ \omega _C< (1-a_w) \ k_{main} \end{array}\right. } \\ f_{H,POL \ scav}= &  \min \{\phi _{SOC,H,2},\phi _{POL};\phi _{aw,3}\}, \\ f_{H,CDB \ scav}= &  \min \{\phi _{SOC,H,2},\phi _{CDB};\phi _{aw,3}\}, \\ f_{H,NDB \ scav}= &  \min \{\phi _{N,H,2},\phi _{NDB};\phi _{aw,3}\}, \\ f_{H,uptake}= &  \min \{\phi _{SOC,H,2},\phi _{TOA};\phi _{aw,3}\}, \\ f_{H,resp}= &  \min \{\phi _{ATP,H,2};\phi _{SOC,H,1};\phi _{aw,3}\}, \\ f_{H,growth}= &  \min \{\phi _{ATP,H};\phi _{SOC,H,1};\phi _{N,H,1};\phi _{pH}\}, \\ f_{H,main}= &  {\left\{ \begin{array}{ll} f^*_{main}, & \text {if}\ Q_{ATP,H}>0 \\ f^*_{main}, & \text {if}\ Q_{ATP,H}=0, \ \omega _H \ge (1-a_w) \ k_{main} \\ \omega _H, & \text {if}\ Q_{ATP,H}=0, \ \omega _H < (1-a_w) \ k_{main} \end{array}\right. } \end{aligned}$$

where

$$\begin{aligned} \omega _H= &  \frac{38}{6} \ r_{H,resp}{M_H}, \\ f_{d,H}= &  {\left\{ \begin{array}{ll} 0, & \text {if}\ Q_{ATP,H}>0 \\ 0, & \text {if}\ Q_{ATP,H}=0, \ \omega _H \ge (1-a_w) \ k_{main} \\ k_d \ (1-a_w) \ (1-\gamma _H), & \text {if}\ Q_{ATP,H}=0, \ \omega _H <(1-a_w) \ k_{main} \end{array}\right. } \end{aligned}$$

with

$$\begin{aligned} \phi _{ATP,C,1}= &  \frac{Q_{ATP,C}}{Q_{max,ATP}}, \\ \phi _{NADPH,1}= &  \frac{Q_{NADPH,C}}{Q_{max,NADPH}}, \\ \phi _{SOC,C,1}= &  \frac{Q_{SOC,C}}{Q_{max,SOC}}, \\ \phi _{N,C,1}= &  \frac{Q_{N,C}}{Q_{max,N}}, \\ \phi _{ATP,C,2}= &  \frac{Q_{\max ,ATP}-Q_{ATP,C}}{Q_{\max ,ATP}}, \\ \phi _{NADPH,2}= &  \frac{Q_{\max ,NADPH}-Q_{NADPH,C}}{Q_{\max ,NADPH}}, \\ \phi _{SOC,C,2}= &  \frac{Q_{max,SOC}-Q_{SOC,C}}{Q_{max,SOC}}, \\ \phi _{N,C,2}= &  \frac{Q_{max,N}-Q_{N,C}}{Q_{max,N}}, \\ \phi _{I,1}= &  \left( \frac{I}{I_{opt}}\right) ^\eta \ e^{\big (1-\frac{I}{I_{opt}}\big )^\eta }, \\ \phi _{I,2}= &  {\left\{ \begin{array}{ll} \big (\frac{I}{I_{opt}}\big )^\eta \ e^{\big (1-\frac{I}{I_{opt}}\big )^\eta }, & \text {if}\ I \le I_{opt} \\ 1, & \text {if}\ I>I_{opt} \end{array}\right. } \\ \phi _{I,3}= &  {\left\{ \begin{array}{ll} 1, & \text {if}\ I>0 \\ 0, & \text {if}\ I=0 \end{array}\right. } \\ \phi _{I,4}= &  {\left\{ \begin{array}{ll} 0, & \text {if}\ I>0 \\ 1, & \text {if}\ I=0 \end{array}\right. } \\ \phi _{aw,1}= &  {\left\{ \begin{array}{ll} \frac{a_w-a_w^{c,1}}{1-a_w^{c,1}}, & \text {if}\ a_w>a_w^{c,1} \\ 0, & \text {otherwise}\ \end{array}\right. } \\ \phi _{aw,2}= &  {\left\{ \begin{array}{ll} \frac{1-a_w}{1-a_w^{c,1}}, & \text {if}\ a_w>a_w^{c,1} \\ \frac{a_w-a_w^{c,2}}{a_w^{c,1}-a_w^{c,2}}, & \text {if}\ a_w^{c,2}<a_w<a_w^{c,1} \\ 0, & \text {otherwise}\ \end{array}\right. } \\ \phi _{aw,3}= &  {\left\{ \begin{array}{ll} 1, & \text {if}\ a_w>a_w^{c,1} \\ \frac{a_w-a_w^{c,2}}{a_w^{c,1}-a_w^{c,2}}, & \text {if}\ a_w^{c,2}<a_w<a_w^{c,1} \\ 0, & \text {otherwise}\ \end{array}\right. } \\ \phi _{DIC}= &  \frac{S_{DIC}}{K_{DIC}+S_{DIC}}, \\ \phi _{pH}= &  exp\bigg (-3 \bigg (\frac{pH-pH_U}{pH_U-pH_L}\bigg )^2\bigg ), \\ \phi _{TOA}= &  \frac{S_{TOA}}{K_{TOA}+S_{TOA}}, \\ \phi _{POL}= &  \frac{\frac{M_{POL}}{M}}{K_{scav,c}+\frac{M_{POL}}{M}}, \\ \phi _{CDB}= &  \frac{\frac{M_{CDB}}{M}}{K_{scav,c}+\frac{M_{CDB}}{M}}, \\ \phi _{NDB}= &  \frac{\frac{M_{NDB}}{M}}{K_{scav,n}+\frac{M_{NDB}}{M}}, \\ \phi _{ATP,H}= &  \frac{Q_{ATP,H}}{Q_{max,ATP}}, \\ \phi _{ATP,H,2}= &  \frac{Q_{max,ATP}-Q_{ATP,H}}{Q_{max,ATP}}, \\ \phi _{SOC,H,1}= &  \frac{Q_{SOC,H}}{Q_{max,SOC}}, \\ \phi _{SOC,H,2}= &  \frac{Q_{max,SOC}-Q_{SOC,H}}{Q_{max,SOC}}, \\ \phi _{N,H,1}= &  \frac{Q_{N,H}}{Q_{max,N}}, \\ \phi _{N,H,2}= &  \frac{Q_{max,N}-Q_{N,H}}{Q_{max,N}}, \\ \phi _{pH}= &  exp\bigg (-3 \bigg (\frac{pH-pH_U}{pH_U-pH_L}\bigg )^2\bigg ). \end{aligned}$$

Here $Q_{max,ATP}$ is the maximum quota for ATP; $Q_{max,NADPH}$ is the maximum quota for NADPH; $a_w^{c,1}$ is the water activity value at which non-cyclic photophosphorylation is inhibited; $a_w^{c,2}$ is the water activity value at which any ATP production is inhibited; $K_{DIC}$ is the half-saturation coefficient of *DIC* on photosynthesis; $I_{opt}$ is the optimum light intensity; $\eta$ is the coefficient of adaptability to non-optimal light conditions; $Q_{max,SOC}$ is the maximum quota for stored organic carbon; $Q_{max,N}$ is the maximum quota for *N*; $pH_U$ and $pH_L$ are *pH* levels at which the organisms are not inhibited, and at which inhibition is complete, respectively; $K_{TOA}$ is the half-saturation constant of organic acids uptake; $K_{scav,c}$ and $K_{scav,n}$ are the half-saturation constant of particulate carbon and nitrogen material, respectively; $k_{main}$ is the maintenance energy requirement in a completely dehydrated SAB; $k_d$ is the decay rate in a completely dehydrated SAB.

Liquid-phase concentrations of volatile compounds, such as oxygen O$_2$ and carbon dioxide CO$_2$, are estimated to be at the steady state using Henry’s law

59 $$\begin{aligned} S_{i,sat}=P_{i} \ k_{H,i}(T), \ i\in \{CO_2,O_2\}, \end{aligned}$$

where $P_{i}$ are the steady-state gas phase partial pressures of components *i*, and

60 $$\begin{aligned} k_{H,i}= k_{H,i,25} \ exp\bigg (\frac{\Delta H^0_{i}}{R}\bigg (\frac{1}{273.15+T_s}-\frac{1}{298.15}\bigg )\bigg ), \ i\in \{CO_2,O_2\}, \end{aligned}$$

where $k_{H,i,25}$ is the Henry’s law coefficients at $25 ^{\circ }C, \Delta H^0_{i}$ is the heat of reaction at standard temperature and pressure, *R* is the universal gas constant and $T_s$ is the stone temperature, representative of the SAB temperature.

SAB pH level is tracked through the charge neutrality condition

61 $$\begin{aligned} \sum _{i}{S_{i,ions}} + S_{OH^-} + S_{H^+} = 0, \end{aligned}$$

where

62 $$\begin{aligned} \sum _{i}{S_{i,ions}}= S_{Cat} + S_{An} - S_{HCO^-_3} - \frac{S_{OA^-}}{2}, \end{aligned}$$

and

63 $$\begin{aligned} S_{OH^-} = \frac{K_{a,H2O}}{S_{H^+}}. \end{aligned}$$

Here $K_{a,H2O}$ is the dissociation constant autoprotolysis of water, $S_{H^+}$ and $S_{OH^-}$ denote the concentrations of hydrogen ions and hydroxide ions dissolved in water. We consider glycolic acid to be representative of the organic acids released by photorespiration (El Moustaid et al. 2017). Note that, then, charge density $S_{OA^-}$ is divided by two: anion glycolic acid has a single negative charge while it is constituted by two moles of carbon. It follows from (61) and (63) that

64 $$\begin{aligned} S_{H^+} = 0.5\left( \sqrt{\bigg (\sum _{i}{S_{i,ions}}\bigg )^2 + 4 K_{a,H2O}}-\sum _{i}{S_{i,ions}}\right) , \end{aligned}$$

with, then,

65 $$\begin{aligned} pH = -log_{10} S_{H^+}. \end{aligned}$$

We estimate SAB water activity using the physically-based method presented in Tenore et al. (2023a), outlined here. The tendency of water to move from one area to another is quantified by water potential, defined as the potential energy of water per unit volume relative to pure water in reference conditions. The water potential of water vapor in air can be expressed as Potts (1994):

66 $$\begin{aligned} \psi _{vap} = \frac{R T}{\bar{V}_w}ln RH, \end{aligned}$$

where *T* is air temperature, $\bar{V}_w$ is the partial molal volume of water and *RH* is the relative humidity of air, defined as

67 $$\begin{aligned} RH = \frac{P_{wv}}{P_{sat}(T)}, \end{aligned}$$

where $P_{wv}$ is the water vapor partial pressure and $P_{sat}(T)$ is the equilibrium water vapor saturation pressure over pure water, computed through the Magnus formula:

68 $$\begin{aligned} P_{sat}(T) = a e^{\big (\frac{b T}{c+T}\big )}, \end{aligned}$$

with *T* expressed in $^\circ$C and coefficients *a*, *b* and *c* determined experimentally.

Assuming the SAB as a mixed biofilm-liquid layer, the SAB water potential can be defined as:

69 $$\begin{aligned} \psi _{SAB} = \frac{R \ T}{\bar{V}_w}ln \ a_w, \end{aligned}$$

where *T* is the SAB temperature. At steady-state, water potential of water vapor $\psi _{vap}$ matches water potential $\psi _{SAB}$ of SAB at the atmosphere-SAB interface, where $T=T^*$. Then, by equating equations (66) and (69) for $T = T^*$, and assuming the water content in the ambient air to be the same as that adjacent to the stone (so that water vapor partial pressure $P_{wv}$ is also the same), that is

70 $$\begin{aligned} RH_a e^{\big (\frac{b T_a}{c+T_a}\big )} = RH (T^*) e^{\big (\frac{b T^*}{c+T^*}\big )}, \end{aligned}$$

water activity $a_w$ can be determined as follows:

71 $$\begin{aligned} a_w = RH_a e^{\big (\frac{b T_a}{c+T_a}\big )}e^{\big (-\frac{b T^*}{c+T^*}\big )}. \end{aligned}$$

This procedure enables the calculation of the SAB water activity from Eq. (71), based on air and stone temperatures as well as humidity conditions.

## Model parameters

Yield parameters in Eqs. 44–58 are determined based on biochemical reactions and stoichiometric considerations, assuming the biomass for both cyanobacteria and heterotrophs to be in the form CN$_{1.7}$O$_{0.5}$N$_{0.2}$. Other parameter values are detailed in Tables 3 and 4, along with relevant literature references. Physical and chemical parameters values (refer to Table 3) are well characterized, being extensively documented in the literature. On the other hand, taxonomy, variability of metabolic pathways related to environmental conditions, and the absence of standardized measurement units contribute to a higher uncertainty associated with most biological parameters (listed in Table 4), making their determination more uncertain. When applicable, biological parameters are derived from literature values, converted using (Bratbak 1985; Sathyendranath et al. 2009):

**Table 1:** Initial values

Initial values	Definition	Unit	Value
$M_{0}$	Initial total mass of SAB solid-phase components	$\textrm{mol C}$	$1.25 \times 10^{-7}$
$M_{C,0}$	Initial cyanobacteria functional biomass	$\textrm{mol C}$	$0.82 M_{0}$
$M_{H,0}$	Initial heterotrophic functional biomass	$\textrm{mol C}$	$0.008 M_{0}$
$M_{POL,0}$	Initial polysaccharides	$\textrm{mol C}$	$0.008 M_{0}$
$M_{CDB,0}$	Initial carbon decayed biomass	$\textrm{mol C}$	0
$M_{NDB,0}$	Initial nitrogen decayed biomass	$\textrm{mol N}$	0
$Q_{SOC,C,0}$	Initial stored organic carbon in cyanobacteria	$\textrm{mol C}/\textrm{mol C}^{-1}$	0.2
$Q_{N,C,0}$	Initial bound nitrogen in cyanobacteria	$\textrm{mol N}/\textrm{mol C}^{-1}$	0.1
$Q_{ATP,C,0}$	Initial ATP in cyanobacteria	$\textrm{mol ATP}/\textrm{mol C}^{-1}$	$5.5 \times 10^{-5}$
$Q_{NADPH,C,0}$	Initial NADPH in cyanobacteria	$\textrm{mol NADPH}/\textrm{mol C}^{-1}$	$10^{-6}$
$Q_{SOC,H,0}$	Initial stored organic carbon in heterotrophs	$\textrm{mol C}/\textrm{mol C}^{-1}$	0.2
$Q_{N,H,0}$	Initial bound nitrogen in heterotrophs	$\textrm{mol N}/\textrm{mol C}^{-1}$	0.1
$Q_{ATP,H,0}$	Initial ATP in heterotrophs	$\textrm{mol ATP}/\textrm{mol C}^{-1}$	$5.5 \times 10^{-5}$
$S_{TOA,0}$	Initial concentration of total organic acids	$\textrm{mol C}/\textrm{m}^3$	0
$S_{OA^-,0}$	Initial concentration of anion organic acids	$\textrm{mol C}/\textrm{m}^3$	0
$S_{HCO^-_3,0}$	Initial concentration of bicarbonate	$\textrm{mol C}/\textrm{m}^3$	0.008

**Table 2:** Environmental variables

Parameter	Definition	Unit
$T_a$	Air temperature	$^{\circ }\textrm{C}$
$T_s$	Stone temperature	$^{\circ }\textrm{C}$
$RH_a$	Relative humidity of air	%
*I*	Light intensity	$\textrm{kmol} \ \textrm{m}^{-2} \ \textrm{d}^{-1}$

**Table 3:** Biological model parameters

Parameter	Definition	Unit	Value	Ref
$k_{nc}$	Max non-cyclic photophosphorylation rate	$\textrm{mol O}_2 \ \textrm{mol C}^{-1} \ \textrm{d}^{-1}$	13.5	Luimstra et al. (2018), Sathyendranath et al. (2009)
$k_{cy}$	Basal cyclic photophosph. rate for $a_w=1$	$\textrm{mol ATP} \ \textrm{mol C}^{-1} \ \textrm{d}^{-1}$	0.61	Jeanjean et al. (1993)
$k_{fix}$	Max carbon fixation rate for cyanobacteria	$\textrm{d}^{-1}$	4	(a)
$k_{growth,C}$	Max growh rate for cyanobacteria	$\textrm{d}^{-1}$	0.84	Tilzer (1987)
$k_{growth,H}$	Max growh rate for heterotrophs	$\textrm{d}^{-1}$	1.07	Rittmann et al. (1986)
$k_{resp}$	Max respiration rate	$\textrm{mol O}_2 \ \textrm{mol C}^{-1} \ \textrm{d}^{-1}$	2	Zavřel et al. (2017)
$k_{Nfix}$	Max nitrogen fixation rate	$\textrm{mol N} \ \textrm{mol C}^{-1} \ \textrm{d}^{-1}$	0.4	Grover et al. (2019), Pinzon and Ju (2006)
$k_{main}$	Maintenance demand in a dehydrated SAB	$\textrm{mol ATP} \ \textrm{mol C}^{-1} \ \textrm{d}^{-1}$	11.70	Gustafsson et al. (1993)
$k_{pol}$	Maximum polisaccharides production rate	$\textrm{d}^{-1}$	0.3	Polizzi et al. (2017)
$k_{scav}$	Maximum scavenging rate	$\textrm{d}^{-1}$	0.17	Merkey et al. (2009)
$k_{uptake}$	Maximum uptake rate	$\textrm{d}^{-1}$	32.4	El Moustaid et al. (2017)
$k_d$	Decay rate in a completely dehydrated SAB	$\textrm{d}^{-1}$	0.01	(a)
$\Delta k_{cy}$	Max increment of cyclic photophosphorylation rate	$\textrm{mol ATP} \ \textrm{mol C}^{-1} \ \textrm{d}^{-1}$	12.9	Jeanjean et al. (1993)
$Q_{max,ATP}$	Maximum quota for ATP	$\textrm{mol ATP} \ \textrm{mol C}^{-1}$	$10^{-4}$	Greiner and Glonek (2021), Mempin et al. (2013), Bratbak (1985)
$Q_{max,NADPH}$	Maximum quota for NADPH	$\textrm{mol NADPH} \ \textrm{mol C}^{-1}$	$10^{-5}$	Tanaka et al. (2021)
$Q_{max,SOC}$	Maximum quota for *SOC*	$\textrm{mol C} \ \textrm{mol C}^{-1}$	2.5	(a)
$Q_{max,N}$	Maximum quota for *N*	$\textrm{mol N} \ \textrm{mol C}^{-1}$	0.5	Grover et al. (2019)
$K_{DIC}$	Half-saturation coeff. of *DIC* on photosynthesis	$\textrm{mol C} \ \textrm{m}^{-3}$	0.1	Wolf et al. (2007)
$K_{scav,c}$	Half-saturation coeff. of carbon particulate material for *H*	$\textrm{mol C} \ \textrm{mol C}^{-1}$	0.03	Lu et al. (2001)
$K_{scav,n}$	Half-saturation coeff. of nitrogen particulate material for *H*	$\textrm{mol N} \ \textrm{mol C}^{-1}$	0.0003	(a)
$K_{TOA}$	Half-saturation coeff. of organic acids uptake	$\textrm{mol C} \ \textrm{m}^{-3}$	0.01	Vincent et al. (2024)
$I_{opt}$	Optimum light intensity	$\textrm{kmol} \ \textrm{m}^{-2} \ \textrm{d}^{-1}$	0.00864	Tenore et al. (2023b)
$\eta$	Coeff. of adaptability to non-optimal light for *C*	−	0.6	Tenore et al. (2023b)
$\gamma$	Inverse specificity factor	$\textrm{mol C} \ \textrm{mol O}_2^{-1}$	0.01	El Moustaid et al. (2017)
$pH_U$	*pH* levels at which the organisms are not inhibited	−	7	Baldanta et al. (2023), Nayak and Prasanna (2007)
$pH_L$	*pH* levels at which inhibition is complete	−	4	Brock (1973)
$a_w^{c,1}$	$a_w$ Threshold for non-cyclic photophosph	−	0.8	(b)
$a_w^{c,2}$	$a_w$ Threshold for ATP production	−	0.6	(b)
$\rho$	Constant microbial density in the SAB	$\textrm{mol C}\ \textrm{m}^{-3}$	500	(a)

(a) This study (b) In the range of values reported in Lange et al. (1994), Wierzchos et al. (2012), Villa et al. (2015)

**Table 4:** Physical and chemical model parameters

Parameter	Definition	Unit	Value	Ref
$P_{CO_2}$	Steady-state gas phase partial pressure of CO$_2$	$\textrm{atm}$	0.00039	Green and Perry (2008)
$P_{O_2}$	Steady-state gas phase partial pressure of O$_2$	$\textrm{atm}$	0.21	Green and Perry (2008)
$k_{H,CO_2,25}$	Henry’s law coefficient at $25 ^{\circ }C$	$\textrm{mol} \ \textrm{atm}^{-1} \ \textrm{L}^{-1}$	$3.4 \times 10^{-2}$	Green and Perry (2008)
$k_{H,O_2,25}$	Henry’s law coefficient at $25 ^{\circ }C$	$\textrm{mol} \ \textrm{atm}^{-1} \ \textrm{L}^{-1}$	$1.4 \times 10^{-3}$	Green and Perry (2008)
$\Delta H^0_{CO_2}$	Heat of reaction at standard temperature and pressure	$\textrm{J} \ \textrm{mol}^{-1}$	25100	Green and Perry (2008)
$\Delta H^0_{O_2}$	Heat of reaction at standard temperature and pressure	$\textrm{J} \ \textrm{atm}^{-1}$	6810	Green and Perry (2008)
$K_{a,H_2O}$	Dissociation constant autoprotolysis of water	−	$10^{-7}$	Green and Perry (2008)
$k_{AB,CO_2}$	Rate constant of CO$_2$ hydrolysis	$\textrm{d}^{-1}$	$2.221 \times 10^{3}$	Rosén and Jeppsson (2006)
$k_{AB,OA}$	Rate constant of OA hydrolysis	$\textrm{m}^{3} \ \textrm{mol}^{-1}\textrm{d}^{-1}$	$10^{7}$	Rosén and Jeppsson (2006)
$K_{a,CO_2}$	Dissociation constant of CO$_2$ hydrolysis	−	$10^{-6.36}$	Green and Perry (2008)
$K_{a,OA}$	Dissociation constant of OA hydrolysis	−	$10^{-3.8}$	Green and Perry (2008)
$\lambda$	SAB thickness	$\upmu$m	15	Tenore et al. (2023a)
$\delta$	Thickness of the thermal boundary layer	$\textrm{cm}$	1	Tenore et al. (2023a)
$k_a$	Thermal diffusivity of air	$\textrm{m}^{2} \ \textrm{s}^{-1}$	$1.9 \times 10^{-5}$	Shen et al. (1998)
$k_b$	Thermal diffusivity of SAB	$\textrm{m}^{2} \ \textrm{s}^{-1}$	$1.47 \times 10^{-7}$	Pinel et al. (2021)
*a*	Magnus formula coefficient	$\textrm{Pa}$	610.94	Lawrence (2005)
*b*	Magnus formula coefficient	−	17.625	Lawrence (2005)
*c*	Magnus formula coefficient	$^\circ \textrm{C}$	243.04	Lawrence (2005)
*R*	Universal gas constant	$\textrm{J} \ \textrm{mol}^{-1} \ ^\circ \textrm{K}^{-1}$	8.314	Green and Perry (2008)
$\bar{V}_w$	Partial molal volume of water	$\textrm{m}^{3} \ \textrm{mol}^{-1}$	$18 \times 10^{-6}$	Potts (1994)

72 $$\begin{aligned} \mathrm {Mass \, of \, carbon \, per \, cell \, volume }= &  5.6 {\cdot } 10^{-13} \textrm{g} / \mu \textrm{m}^3, \end{aligned}$$

73 $$\begin{aligned} \mathrm {Carbon \, to \, chlorophyll \, ratio}= &  100 \, \textrm{g}/\textrm{g}. \end{aligned}$$

However, the determination of some biological parameter values requires additional considerations. We attempt to use reasonable, though admittedly rough, estimates. The decay rate is set an order of magnitude lower than literature values for microorganisms in immersed biofilms, accounting for the specialized strategies of SAB microorganisms in responding to stress conditions, such as dormancy mechanisms. The same choice is made for microbial density, considering SABs to be less densely packed than immersed biofilms (which is consistent with our own observations). Two water activity thresholds $a_w^{c,1}$ and $a_w^{c,2}$ are set within the range of values provided in the literature, allowing for the assessment of different sensitivities of metabolic processes to water stress conditions (e.g., cyclic and non-cyclic photophosphorylation). The inverse specificity factor for photorespiration $\gamma$ is set, conservatively, at the lower limit of the literature survey range provided in El Moustaid et al. (2017), supposing subaerial cyanobacteria to be well-adapted to significant presence of oxygen. The maximum quota of stored organic carbon is set to be 5-fold higher than the maximum content of bound nitrogen, aligning with the biomass composition (CN$_{1.7}$O$_{0.5}$N$_{0.2}$). The optimal pH parameter $pH_U$ is set at 7 as cyanobacteria are generally reported to prefer neutral pH (Baldanta et al. 2023; Nayak and Prasanna 2007), though it is possible that SAB cyanobacteria take different values. Conversely, the inhibition limit $pH_L$ is, conservatively, set at 4, representing the pH threshold below which cyanobacteria typically cannot survive, as documented in Brock (1973).

Though we have not attempted a general sensitivity analysis due to the large number of parameters, a few parameters seem to stand out in importance. In particular, results appear to be sensitive to the water activity thresholds $a_w^{c,1}$ and $a_w^{c,2}$, which regulate the maintenance demand and all metabolic processes related to ATP and NADPH production, as well as carbon and nitrogen supply. This is not surprising, since environmental data suggests that water activity conditions are frequently marginal, and related stresses are thought to be important for SAB microorganisms. The model agrees with and reinforces the general belief on this point.

In addition, model results are relatively strongly affected by the maximum rate at which carbon is fixed $k_{fix}$. Indeed, carbon fixation by cyanobacteria (or other phototrophs) is essential for the development of phototrophic SABs as it allows for the accumulation of organic carbon within the ecosystem, supporting its productivity and stability. In subaerial environments, optimal light and water conditions for carbon fixation are often limited to short periods of time; consequently, the rate at which this process occurs can become the discriminating factor for the successful development of SAB. Again, this is consistent with general belief—SAB cyanobacteria are thought to be able to respond quickly and efficiently to improvements in water conditions.

Lastly, though the model does not predict pH variations to be significant in mixed species SABs, it is still of interest because of the possibility of interactions between SAB and the stone substratum. Parameters $k_{fix}$, along with microbial density $\rho$, and the inverse specificity factor for photorespiration $\gamma$, play an important role in regulating pH, and are rather uncertain (though $\rho$ is a likely target for measurement). $k_{fix}$ and $\gamma$ impact the rate of photorespiration and the related acid production, and $\rho$ affects the amount of organic acids produced per unit volume. Small variations of these parameters may significantly affect the overall concentration of non-volatile acids, thus impacting the predicted pH level and metabolic dynamics. Additionally, pH is constrained by the dissociation constant of the equilibrium reaction of organic acids, which is approximately $10^{-3.8}$. In other words, for pH values below 3.8, the photorespiration process will lead mainly to the formation of acids in their non-ionized form, which will not further lower the pH. In light of this, in the absence of heterotrophs, the eventual development and proliferation of SABs will depend essentially on the viable pH range, that is on $pH_U$ and, particularly, on $pH_L$. Finally, the chemical composition and reactivity of the stone, including its pKa value, might potentially constrain the pH level within the ecosystem, thereby affecting its overall dynamics.
