# Supplementary material for: A Mathematical Model of Diel Activity and Long Time Survival in Phototrophic Mixed-Species Subaerial Biofilms
Source: Bull Math Biol. 2024 Aug 28;86(10):123. doi: 10.1007/s11538-024-01348-3 (PMC11358337; doi:10.1007/s11538-024-01348-3)
Supplement: Supplementary file 1 — (pdf 1710 KB) [file 11538_2024_1348_MOESM1_ESM.pdf]

Supplementary material

A. Tenore[1*], F. Russo[1], J. Jacob[2], J.D. Grattepanche[3], B. Buttaro[4], I. Klapper[5]

[1*]Department of Mathematics and Applications, University of Naples Federico II, Naples, Italy.
[2]U.S. National Park Service, North Atlantic-Appalachian Region, Historic Architecture, Conservation, and Engineering Program, United States of America.
[3]Department of Biology, Temple University, Philadelphia, PA, United States of America.
[4]Sol Sherry Thrombosis Research Center, Katz School of Medicine, Temple University, Philadelphia, PA, United States of America.
[5]Department of Mathematics, Temple University, Philadelphia, PA, United States of America.

*Corresponding author(s). E-mail(s): alberto.tenore@unina.it; Contributing authors: fabiana.russo@unina.it; judy_jacob@nps.gov; jean-david.grattepanche@temple.edu; bettina.buttaro@temple.edu; klapper@temple.edu;

Numerical Study S1 - Representative case at Federal Hall National Memorial

Here we report a case study based on the environmental profile for a specific summer day at Federal Hall National Memorial, using the same model and methods as in the main text. Fig. 1 illustrates the environmental input variables (top) and the computed water activity (bottom). Water activity trends are similar to those observed in the representative study at Jefferson Memorial (Section 4.2.1), except during the late hours of darkness, when water activity at Federal Hall reaches very high values (exceeding 0.9) due to high humidity levels. Furthermore, the urban canyon environment of Federal Hall results in reduced light intensity and ambient air vs stone temperatures. Similar to numerical study 1 (Section 4.2.1), we used the data for this same summer day,

repeated periodically, to simulate until an approximately periodic state was attained. Numerical results for the final day of the multi-day simulation are depicted in Figs. 2-4.

As evident from these figures, SAB metabolic patterns resemble those predicted for the Thomas Jefferson Memorial SAB. Once again, the model predicts metabolic activity to occur within specific time intervals of the day depending on water activity and light availability. Increased water activity levels in the early morning promote relatively intense metabolic processes including photosynthesis, ATP and NADPH production, and biomass synthesis. Later, water activity diminishes during midday as temperatures rise and humidity levels drop. As a result, metabolic activity becomes limited, maintenance demands increase, and decay occurs. The main differences with the results predicted in the case study at the Thomas Jefferson Memorial are observed during final darkness hours when water activity peaks at Federal Hall National Memorial. This elevated water availability leads to limited maintenance demand, more easily met by both cyanobacteria and heterotrophs. As a consequence, decay processes do not occur, and an early growth period for heterotrophs before sunrise is observed. This early growth is less pronounced in cyanobacteria, which require light for biosynthesis processes. The SAB composition, reported in Fig. 5, is also similar to that predicted for the Thomas Jefferson Memorial, with a slight difference in the abundance of heterotrophs and polysaccharides: the early growth of heterotrophs during the night results in a higher abundance of heterotrophs and consequently increased consumption (and lower abundance) of polysaccharides.

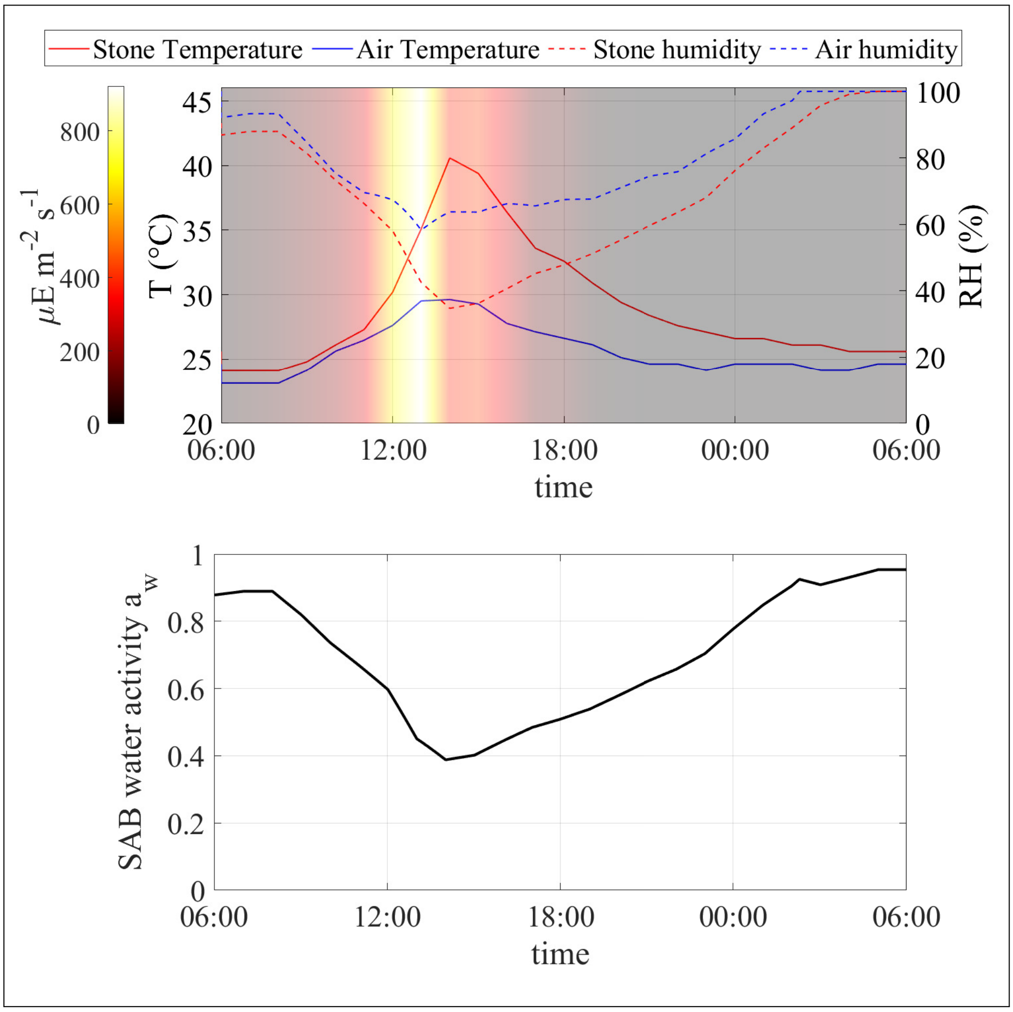

Fig. (1) Numerical study S1 – (top) daily profiles of air and stone temperatures and relative humidities, and light intensity in a summer day at Federal Hall National Memorial, New York City (USA). (Bottom) Computed SAB water activity (at SAB thickness $h = 15\mu$m). (Red solid) stone temperature, (blue solid) ambient air temperature away from the stone surface, (red dash) relative humidity at stone surface, (blue dash) relative humidity in ambient air away from the stone surface, (black solid) computed water activity. Background colors indicate light intensity ($\mu E\ s^{-1}\ m^{-2}$).

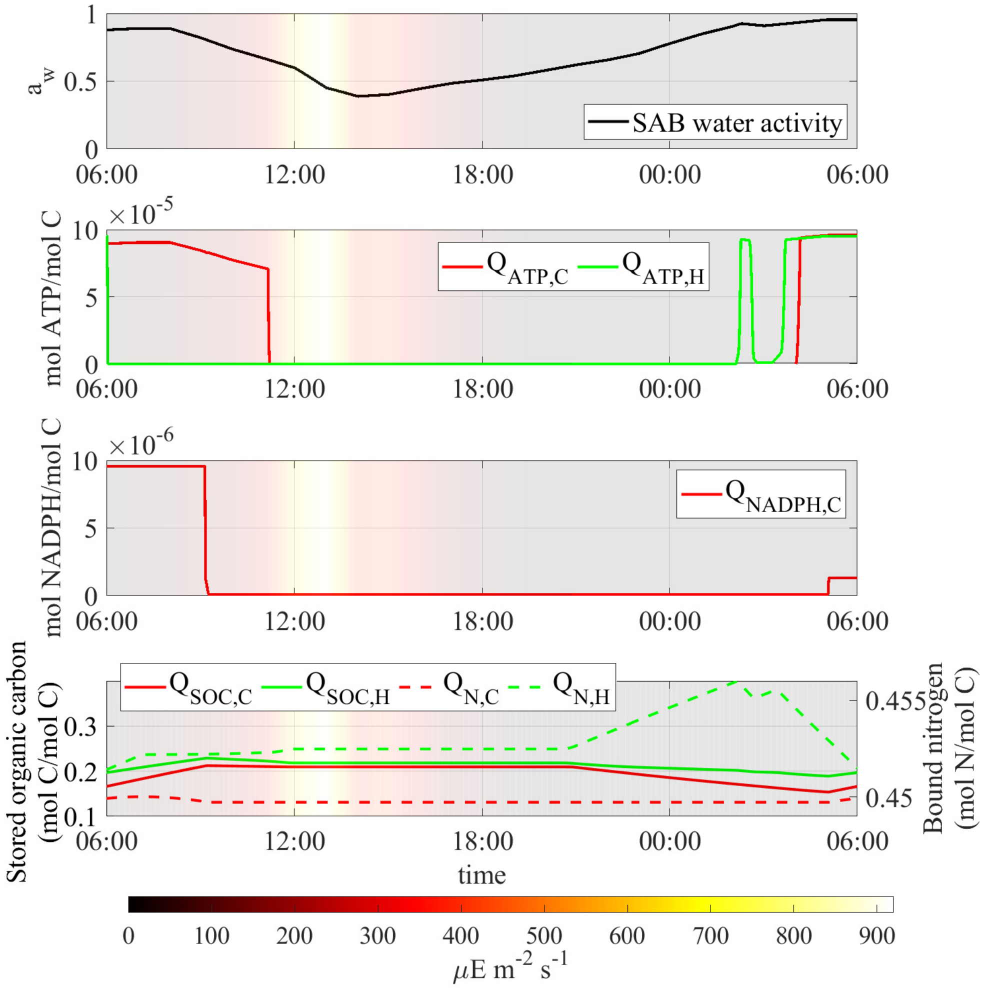

Fig. (2) Numerical study S1 – computed periodic steady-state profiles of SAB water activity, quota of ATP $Q_{ATP,i}$, NADPH Q_{NADPH}, stored organic carbon $Q_{SOC,i}$ and bound nitrogen $Q_{N,i}$. Background colors indicate light intensity ($\mu E\ s^{-1}\ m^{-2}$).

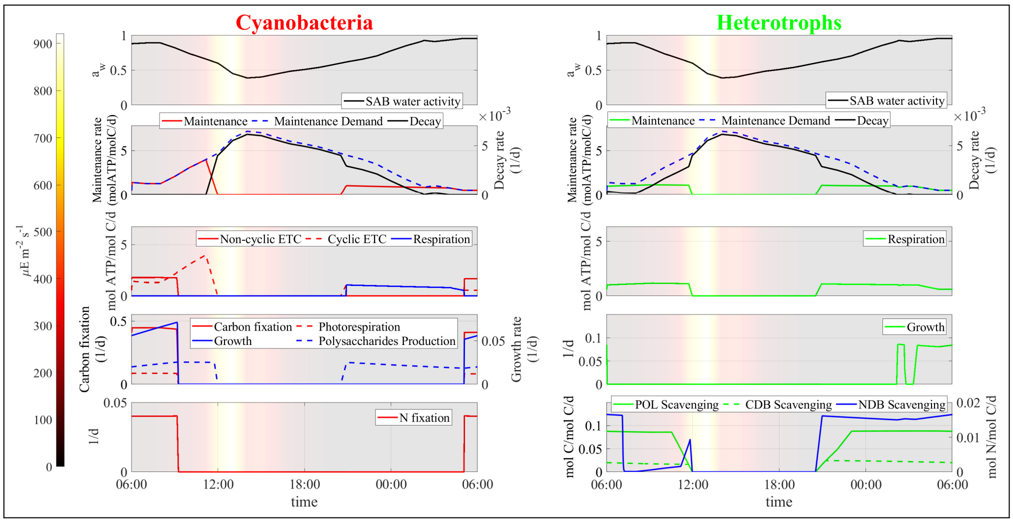

Fig. (3) Numerical study S1 – computed periodic steady-state profiles of SAB water activity, and main metabolic rates in cyanobacteria and heterotrophs. Background colors indicate light intensity ($\mu E\ s^{-1}\ m^{-2}$).

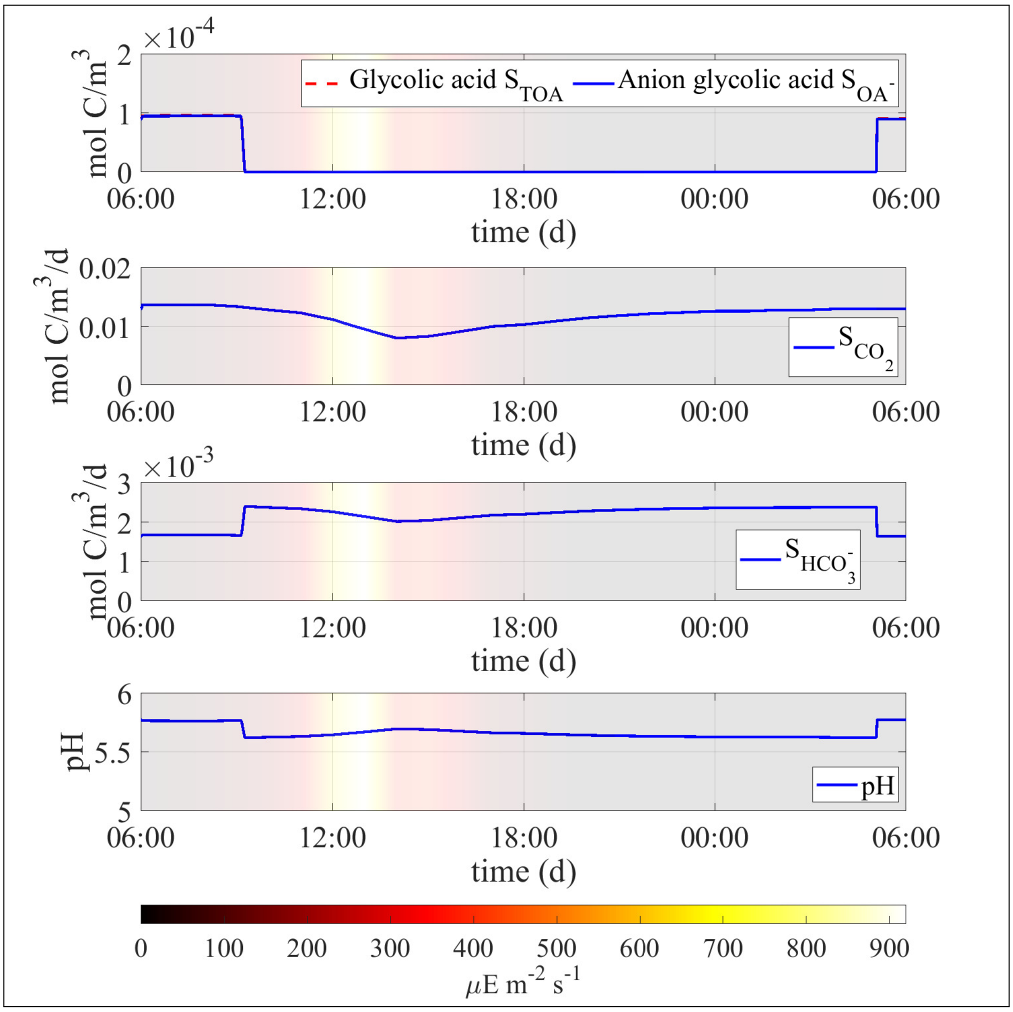

Fig. (4) Numerical study S1 – computed periodic steady-state profiles of dissolved compounds (total and anion glycolic acid, carbon dioxide and bicarbonate), and pH level. Background colors indicate light intensity ($\mu E\ s^{-1}\ m^{-2}$).

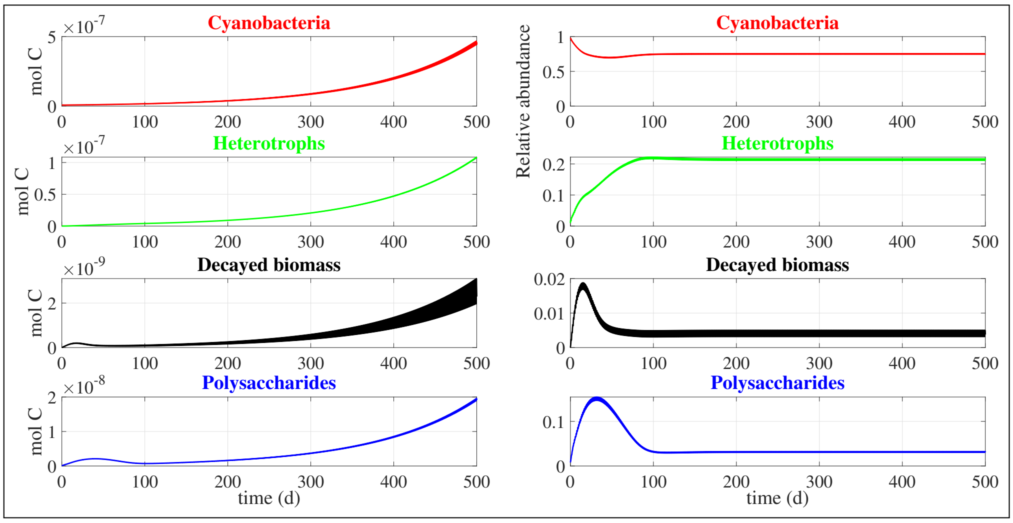

Fig. (5) Numerical study S1 – time evolution of mass (left) and relative abundance (right) of SAB solid-phase components: cyanobacteria ($M_C(1+Q_{SOC,C})$), heterotrophs ($M_H(1+Q_{SOC,H})$), polysaccharides (M_{POL}), decayed biomass (M_{CDB}). Note the different scales on the vertical axes.
